# Target flow deviations on the cardiopulmonary bypass cause postoperative delirium in cardiothoracic surgery—a retrospective study evaluating temporal fluctuations of perfusion data

**DOI:** 10.1093/icvts/ivae016

**Published:** 2024-01-30

**Authors:** Johannes Krefting, Hagen Gorki, Markus Hoenicka, Günter Albrecht, Robert Kraft, Andreas Liebold

**Affiliations:** Department of Cardiothoracic and Vascular Surgery, Ulm University Medical Center, Ulm, Germany; Department of Cardiothoracic and Vascular Surgery, Ulm University Medical Center, Ulm, Germany; Department of Cardiothoracic and Vascular Surgery, Ulm University Medical Center, Ulm, Germany; Department of Cardiothoracic and Vascular Surgery, Ulm University Medical Center, Ulm, Germany; Department of Cardiothoracic and Vascular Surgery, Ulm University Medical Center, Ulm, Germany; Department of Cardiothoracic and Vascular Surgery, Ulm University Medical Center, Ulm, Germany

**Keywords:** Cardiopulmonary bypass, Postoperative delirium, Algorithm-based data processing, Cardiac surgery, Target flow deviations

## Abstract

**OBJECTIVES:**

Postoperative delirium (POD) is common, costly and associated with long-term morbidity and increased mortality. We conducted a cohort study to assess the contribution of cardiopulmonary bypass (CPB) to the development of POD by means of algorithm-based data processing.

**METHODS:**

A database was compiled from 3 datasets of patients who underwent cardiac surgery between 2014 and 2019: intensive care unit discharge files, CPB protocols and medical quality management records. Following data extraction and structuring using novel algorithms, missing data were imputed. Ten independent imputations were analysed by multiple logistic regression with stepwise deletion of factors to arrive at a minimal adequate model.

**RESULTS:**

POD was diagnosed in 456/3163 patients (14.4%). In addition to known demographic risk factors and comorbidities like male sex, age, carotid disease, acute kidney failure and diabetes mellitus, cardiopulmonary parameters like total blood volume at the CPB [adjusted odds ratio (AOR) 1.001; confidence interval (CI) 1.1001–1.002] were independent predictors of POD. Higher values of the minimal blood flow were associated with a lower risk of POD (AOR 0.993; CI 0.988–0.997). Flow rates at least 30% above target did emerge in the minimal adequate model as a potential risk factor, but the confidence interval suggested a lack of statistical significance (AOR 1.819; 95% CI: 0.955–3.463).

**CONCLUSIONS:**

CPB data processing proved to be a useful tool for obtaining compact information to better identify the roles of individual operational states. Strict adherence to perfusion limits along with tighter control of blood flow and acid–base balance during CPB may help to further decrease the risk of POD.

**Figure:**
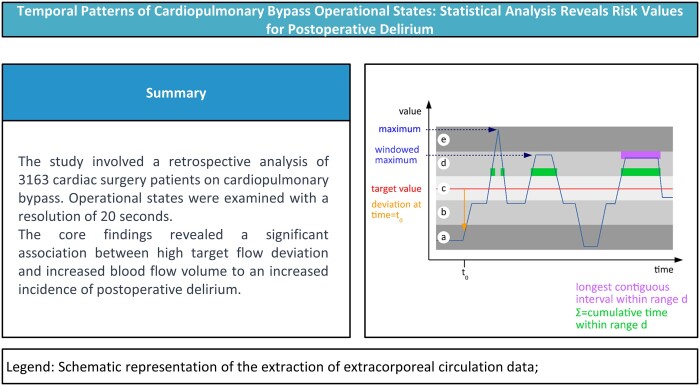

## INTRODUCTION

Postoperative delirium (POD) is a transient neurocognitive disorder in which the patient's awareness, attention and cognitive performance are acutely impaired. It is a common consequence of major surgery in older patients. Both, the average age of cardiac surgery patients and the complexity of the interventions have increased steadily in recent years, causing POD to become more relevant and leading to greater awareness of this serious postoperative concern [1]. As a complication of cardiac surgery, the incidence varies between 10% and 60%, depending on the study and the criteria used to define delirium [2, 3]. POD results in a prolonged stay in the ICU and increasing hospital costs as well as an increase in morbidity and mortality [4]. Although techniques and equipment have evolved significantly over the past 60 years, cardiopulmonary bypass (CPB) is still considered a risk factor for the occurrence of postoperative complications [5]. Blood flow and pressure are usually maintained close to their targets because they are critical for organ perfusion; however, surgical procedures and intraoperative complications may lead to deviations. We hypothesized that the risk of POD may be affected by flow and pressure deviations immediately, or if those deviations persist for a certain contiguous or cumulative time. Similar considerations apply to body temperature, blood pH and blood gas concentrations. These potential mechanisms require an in-depth analysis of perfusion data from the CPB that goes beyond descriptive statistics. We developed a data processing tool that allowed an efficient analysis of the raw perfusion data. The algorithms organized the perfusion data into clinically meaningful value intervals and provided temporal properties of CPB data, such as the longest contiguous time or the total time spent within an interval. These temporal patterns of CPB operational states made the extracorporeal circulation (ECC) amenable for a detailed statistical analysis. These analyses were used to identify risk values of the different CPB parameters prompting POD.

## PATIENTS AND METHODS

### Study design

We conducted a monocentric cohort study to assess the contribution of CPB to the primary end point development of POD (ICD F05.8).

### Ethics

The study complied with the Declaration of Helsinki and was approved by the Ethics Committee of Ulm University (file no. 236/20). The requirement for informed consent was waived by the Ethics Committee of Ulm University as all study data had been collected as part of routine anamnesis, diagnosis and treatment and were accessed retrospectively.

### Inclusion and exclusion criteria

All consecutive patients, who consequently underwent any type of cardiac surgery for any type of pathology (Supplementary Material, Table S1) with the use of a CPB in the Department of Cardiothoracic and Vascular Surgery at Ulm University Medical Center from 1 January 2014 through 31 December 2019, were eligible. Patient records before the starting date were not suitable for the present study as the ICU file format prior to 2014 differed and lacked several demographic parameters and clinical events. The inclusion criterium was the use of a CPB during surgery. Anaesthesia for all patients was administered in line with established protocols, which encompassed the placement of an arterial line, intubation, central venous catheter and urinary catheter. For the initiation of anaesthesia, the agents used were fentanyl, either propofol or etomidate and pancuronium. Maintenance of anaesthesia was managed with sevoflurane and an ongoing infusion of sufentanil up to the point of CPB. Once on CPB, a steady infusion of propofol and sufentanil was utilized. Patients with incomplete CPB data were excluded. CPB data were considered incomplete if they lacked mandatory data for subsequent analysis, such as blood flow or acid–base values that could not be completed by imputation. Duplicates resulting from surgical revisions or readmissions to the ICU, were excluded as well.

### Database construction

**Figure 1:**
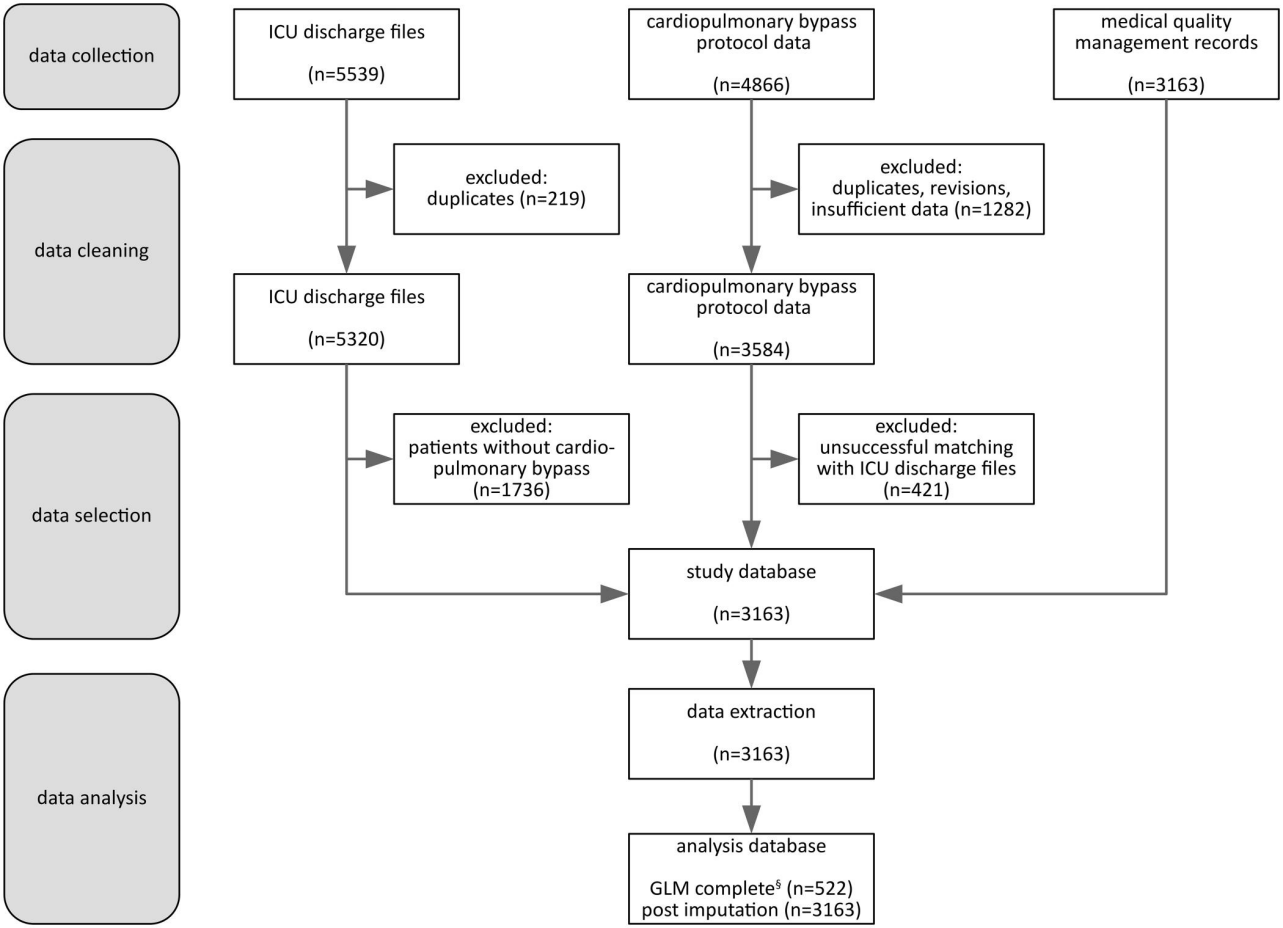
Assembly and analysis of the study data. *n* = number of patients; patient datasets were considered complete if they contained non-Null entries in each of the explanatory variables which were part of the initial generalized linear model (GLM) of postoperative delirium. Multiple imputation was used to fill in missing values which allowed to use all patients of the analysis database for modelling.

The study database was compiled from ICU discharge files, CPB protocols and medical quality management records. The ICU discharge files included postoperative clinical events as well as demographic data. ICD-10 diagnoses of delirium and comorbidities were queried from the local quality management records, which were documented by senior physicians. Delirium assessment by means of the Confusion Assessment Method for the Intensive Care Unit [6] was routinely performed by clinical staff before morning rounds. POD patients were identified based on the ICD-10 code ‘F05.8’ (Delirium not caused by alcohol or other psychotropic substances) in the ICU discharge files or the quality management record. The diagnosis F05.1, Delirium in dementia, was excluded. The CPB protocols contained detailed information about the CPB components used and ECC parameters with a temporal resolution of 20 s. Datasets were matched and compiled into a third normal form relational database (Fig. 1).

### Data cleanup and extraction

Descriptive statistics were calculated for all CPB parameters. The values of the parameters, including blood flow, arterial blood pressure, arterial blood gas data and temperature over time, were analysed to define a set of factors for each parameter. A novel Python-based data processing tool was created to extract all CPB values, process and assign them to predefined value intervals. Ranges were defined by categorizing continuous numerical data along predefined cut points. Along with median, minima and maxima, the following 3 data characteristics describing the complete ECC time of all patients, were calculated:

1. Sustained extrema which were maintained for at least 140 s, referring to extrema which were likely set or tolerated on purpose, as opposed to inadvertent outliers.
2. The longest contiguous time interval spent within a given range, i.e. the duration of the longest single exposure to a particular condition.
3. The cumulative time spent within a given range, i.e. the overall duration of the exposure to a particular condition.

Time intervals and cumulative times were evaluated both as absolute time and as fractions of total CPB time (Supplementary Material, Table S2).

### Statistical analysis

Data were analysed with the software R Version 3.6.3. POD was evaluated as a binary outcome variable. Potential influences of demographic, as well as perioperative explanatory variables on POD, were first assessed in univariable analyses by computing odds ratios with median unbiased estimation and point biserial correlation coefficients. Dependence of POD on sex and age was analysed by analysis of variance and the Wilcoxon rank sum test with Holm correction (Supplementary Material, Table S3 and Supplementary Material, Fig. S1). Significance was assumed for *P* < 0.05. The response variable POD was expressed as a function of explanatory variables with generalized linear models (GLMs) using the logit link function. Factors for the maximal model were selected according to the results of the univariable analysis. Collinearity of explanatory variables was identified with pairs plots and pairwise correlations. Models were simplified by replacing pairs or groups of explanatory variables with pairwise correlations of 0.8 or higher with the variable that showed the highest correlation to POD. To avoid the exclusion of patients from the GLM analyses owing to missingness, multiple imputation [7] was carried out. Ten imputations were calculated and evaluated with GLMs independently. In each imputed dataset, maximal models were simplified stepwise until a minimum of Akaike’s information criterion identified the minimal adequate models. A consensus model was constructed from all factors which were retained in at least one of the 10 minimal adequate models. This consensus model was further simplified stepwise and re-evaluated in all 10 imputations until the arithmetic mean of the 10 Akaike’s information criteria arrived at a minimum. Parameter estimates and their standard deviations, based on the 10 imputations using the final model, were pooled according to Rubin’s rules. The performance of the final model was assessed by calculating McFadden’s *R*-squared value and by preparing receiver-operator characteristics curves.

## RESULTS

A total of 3163 patients with data from all 3 sources were included in the analysis (Fig. 1). POD was documented in 456 (14.4%) patients.

### Demographic parameters

Demographic data and preoperative risk factors are summarized in Table 1. Unadjusted odds ratios and correlation coefficients indicate potential effects on the occurrence of POD of several variables. Age, male sex, acute renal failure, chronic kidney disease, carotid artery disease, diabetes mellitus, right-sided heart failure, atrial fibrillation and prior cardiopulmonary resuscitation were demographic factors and comorbidities with significant positive correlations or unadjusted odds ratios above unity. Weight, body surface area (BSA), obesity, hypertension, familial predisposition and hyperlipidaemia showed significant negative correlations or odds ratios below unity.

**Table 1:** Demographic and preoperative data

	No delirium (*n* = 2707)	Delirium (*n* = 456)	Total (*n* = 3163)	Test statistic	*P*-value
				Point biserial correlation	
Age (years)	66 (58–73)	72 (66–77)	67 (59–74)	0.175	<0.0001
Height (cm)	172 (166–178)	172 (166–178)	172 (166–178)	−0.019	0.29
Weight (kg)	82 (73–93)	80 (71–91)	82 (72–93)	−0.037	0.039
Body mass index (kg/m^2^)	27.7 (24.9–30.9)	27.1 (24.5–30.3)	27.5 (24.8–30.8)	−0.032	0.073
Body surface area (m^2^)	1.96 (1.82–2.10)	1.95 (1.82–2.07)	1.96 (1.82–2.09)	−0.039	0.030
				Odds ratio (95% CI)	
Male sex, *n* (%)	2011 (74.3%)	359 (78.7%)	2370 (74.9%)	1.28 (1.01–1.63)	0.041
Obesity, *n* (%)	604 (22.3%)	81 (17.8%)	685 (21.7%)	0.75 (0.58–0.97)	0.027
Arterial hypertension, *n* (%)	1351 (49.9%)	189 (41.4%)	1540 (48.7%)	0.71 (0.58–0.87)	0.0008
Diabetes mellitus, *n* (%)	566 (20.9%)	131 (28.7%)	697 (22.0%)	1.53 (1.22–1.90)	0.0002
Hyperlipoproteinemia, *n* (%)	1538 (56.8%)	217 (47.6%)	1755 (55.5%)	0.69 (0.57–0.84)	0.0002
Family history of cardiovascular disease, *n* (%)	519 (19.2%)	58 (12.7%)	577 (18.2%)	0.62 (0.46–0.82)	0.0006
Current or previous nicotine abuse, *n* (%)	418 (15.4%)	54 (11.8%)	472 (14.9%)	0.74 (0.54–0.99)	0.042
Chronic kidney disease, *n* (%)	216 (8.0%)	57 (12.5%)	273 (8.6%)	1.65 (1.20–2.24)	0.0024
Carotid artery disease, *n* (%)	191 (7.1%)	49 (10.7%)	240 (7.6%)	1.59 (1.13–2.20)	0.0084
Chronic obstructive pulmonary disease, *n* (%)	187 (6.9%)	34 (7.5%)	221 (7.0%)	1.09 (0.73–1.57)	0.66
Left heart failure, *n* (%)	1317 (48.7%)	241 (52.9%)	1558 (49.3%)	1.18 (0.97–1.44)	0.097
Right heart failure, *n* (%)	48 (1.8%)	25 (5.5%)	73 (2.3%)	3.22 (1.93–5.24)	<0.0001
Peripheral arterial disease, *n* (%)	162 (6.0%)	36 (7.9%)	198 (6.3%)	1.35 (0.91–1.94)	0.13
Pulmonary hypertension, *n* (%)	128 (4.7%)	22 (4.8%)	150 (4.7%)	1.03 (0/63–1.60)	0.91
Atrial fibrillation, *n* (%)	783 (28.9%)	194 (42.5%)	977 (30.9%)	1.82(1.48–2.23)	<0.0001
History of stroke, *n* (%)	29 (1.1%)	5 (1.1%)	34 (1.1%)	1.05 (0.35–2.52)	0.92
History of cardiopulmonary resuscitation, *n* (%)	29 (1.1%)	15 (3.3%)	44 (1.4%)	3.16 (1.63–5.86)	0.0010

The test statistic and *P*-values describe their relationship with the occurrence of postoperative delirium in univariable analyses. Continuous numeric data are given as median (1st quartile to 3rd quartile). All other data report frequencies as patient counts (% of patients within group or total).

CI: confidence interval.

### Perioperative parameters

Non-elective surgery, including emergency and urgent procedures, combined procedures, high CPB blood volume, as well as a long duration of bypass time, cross-clamp time and reperfusion time were positively associated with the development of POD (Supplementary Material, Table S4). Deep cooling had been used in 31 (6.8%) of POD patients and in 105 (3.8%) control patients (*P* = 0.006). Selective cerebral perfusion was applied to 28 (6.1%) of POD patients and to 71 (2.6%) of control patients (*P* = 0.0001). Patients who developed POD had lower minimal blood flow rates and higher sustained maximal flow rates compared to those who did not develop POD (Supplementary Material, Tables S5 and S6). These patients spent more time with flow rates up to 3 l/min and above 4.5 l/min, with higher relative times for flow rates up to 1.5 l/min and above 6 l/min. The longest contiguous time intervals within the flow rate ranges also showed the same pattern. POD patients had longer absolute times spent deviating from their target flows in both directions but spent less time relative to the total CPB time around their target flow. Moreover, both the absolute and relative times spent at higher deviations from the target flow in both directions were longer in POD patients. In summary, patients who developed POD had a pattern of lower minimal flow rates, higher sustained maximal flow rates and longer times spent deviating from their target flows. POD patients had slightly elevated maximal blood pressures and a lower sustained minimum, meaning that their blood pressure readings tended to fluctuate more over the course of CPB. They spent a larger fraction of the total CPB time at low pressures up to 40 mmHg (6.1% vs 5.5%). Total times at all pressure ranges up to 80 mmHg were longer in these patients, reflecting increased CPB times. Longest contiguous times spent below 60 mmHg were higher in POD patients, who spent a lower fraction of CPB time at pressures between 60 and 80 mmHg (20.4% vs 21.8%).

In terms of blood pH and carbon dioxide levels, POD patients had a higher minimal blood pH and spent longer total times and contiguous time intervals at pH values of 7.2. Furthermore, they showed a lower minimal arterial carbon dioxide partial pressure. While POD patients spent shorter contiguous fractions of CPB time at PaCO_2_ from 40 to 60 mmHg they showed a lower minimal PaCO_2_ and longer total and contiguous time intervals at a PaCO_2_ of <40 mmHg. This means that they had higher blood pH and lower carbon dioxide levels, which could be indicative of excess of removal of carbon dioxide through the CPB during low perfusion rates. In summary, our findings suggest that POD patients may experience a different blood pressure and acid–base profile during CPB, which could be relevant to their cognitive outcomes.

POD patients had higher sustained maximal PaO2 levels, with longer total times spent at very low (<70 mmHg) and very high (240 mmHg and above) PaO2 levels. However, they had a lower fraction of total CPB time spent at intermediate PaO2 levels (110–240 mmHg). Similar patterns can be found in terms of continuous time intervals: POD patients spent longer times at very low and very high PaO2 levels, but shorter times at intermediate PaO2 levels (110–140 mmHg), with lower contiguous time fractions between 110 and 240 mmHg.

Overall, POD patients had different patterns of PaO2 levels during CPB, with longer times spent at very low and very high levels, and shorter times at intermediate levels.

Finally, by comparing haematocrit levels between patients with and without POD, no significant differences were found at the extremes of the range (up to 20% and 40% and above). However, in the intermediate ranges (20–30% and 30–40%), patients with POD showed slightly longer exposure times, with statistically significant correlations.

### Multiple regression analysis

In addition to demographic factors and comorbidities, several CPB-related factors were identified as predictors of POD in the multiple regression analysis (Supplementary Material, Table S7). The use of a conventional heart-lung machine instead of a minimally invasive ECC, the surgery being non-elective, the use of Bretschneider cardioplegia solution and longer contiguous time intervals at >30% above target CPB blood flow were part of the minimal adequate model as potential risk factors; however, they were not found to be statistically significant.

The minimum CPB blood flow relative to target flow that was maintained for at least 140 s was an independent predictor of POD. The total CPB blood volume was identified as an independent predictor of POD. Each additional litre of blood increased the risk for POD by a factor of 1.001.

The longest continuous time spent at the interval between 110 and 140 mmHg as well as the absolute time of haematocrit levels being in the interval between 30% and 40% appeared to be protective but did not reach significance in the minimal adequate model. Later years of operation were identified as a risk factor with an adjusted odds ratio (AOR) of 1.084 [95% confidence interval (CI): 1.020–1.151], suggesting a marginal increase in risk with each passing year. The AOR for CPB target flow rates was 0.724 (95% CI: 0.547–0.958), indicating that higher target flows were significantly associated with reduced risk for POD.

The pooled receiver-operating characteristic analysis of the risk of developing POD of the final regression model resulted in an area under the curve of 0.743 (95% CI: 0.719–0.766), indicating an acceptable discrimination (Supplementary Material, Fig. S2). The average McFadden’s *R*-squared value was 0.1081 (standard deviation: 0.0007).

## DISCUSSION

Univariable and multivariable analyses confirmed the detrimental effects of clinically established demographic risk factors and comorbidities, establishing a foundational susceptibility to POD. The novel analysis of functional CPB parameters identified various effects of distinct blood flow, haematocrit and oxygen partial pressure parameters, which may contribute to the progression from susceptibility to manifest POD.

The present study attempted to analyse CPB data in deeper detail to understand which parameters are most relevant as POD determinants. POD patients had longer times for various flow value intervals due to their overall longer ECC times. Data normalized to the total ECC time tended to be similar between groups, except for extremely low or high flows indicating a higher amplitude and greater variability of blood flow values for POD patients. POD patients spent larger fractions of total ECC time at <50% or >130% of the target flow, both in terms of cumulative time and longest contiguous time. The latter proved to be part of the minimal adequate model but failed to reach statistical significance. Notably, POD patients also presented with significantly lower minimal flow values, which emerged as a significant predictor for POD in the minimal adequate model. A low systemic perfusion leads to an insufficient cerebral supply, which may trigger POD. Conversely, hyperperfusion may increase the risk of POD as well. It can be speculated that the extent of inflammation, which is a well-known drawback of CPB, can trigger vasodilation. The need to maintain an adequate mean arterial pressure can lead to hyperperfusion and an increased total blood flow volume in addition to administering vasopressors. High blood flow rates on CPB result in an increased exposure of blood to the artificial surface of the CPB system. This can potentially trigger further systemic inflammation and thus establish a vicious cycle. In addition, higher flow rates increase the speed of microemboli and particles travelling through the circuit, reducing their contact time with defoamer material, and causing more emboli to be discharged into the circulation. Furthermore, a sudden increase in the pump flow rate to achieve full-flow CPB can shorten the blood circulation time between the aortic cannula and carotid arteries, accelerating the speed of air bubbles [8]. This increases the risk of air bubbles reaching the brain before being reabsorbed, thus further contributing to the development of POD. Thudium *et al.* [9] showed that the overall duration of hyperperfusion in patients was associated with the development of delirium. Especially for elder individuals, that exhibited a reduced baseline mean cerebral artery velocity. These observations bolster the hypothesis that CPB-induced hyperperfusion may provide a mechanistic link between advanced age and the incidence of POD.

Total CPB blood flow volume, but not total CPB time or total operation time, was an independent risk factor of POD, again accentuating the critical role of blood volume. Trials have reported either bypass time, or operation time as independent risk factors [10–12]. An increased inflammation was shown for longer CPB times, but not singularly for CPB blood flow [13]. The total CPB blood flow accumulates during the aortic cross-clamp (ACC) time while the key intervention of the operation is performed, best describing the complexity of the surgery and the severity of the underlying disease. Long ACC times lead to a larger amount of blood flow volume through the CPB, which could indirectly indicate unexpected complications or lower expertise of the operating staff. However, this study did not aim in differentiating the reasons for prolonged CPB and ACC times. The question of whether an unexperienced team caused larger total CPB blood flow and therefore more POD must be left unanswered and can be subject to further in-depth studies. In our opinion, the CPB blood flow volume could potentially serve as a surrogate marker of long and/or complex procedures.

In the univariable analysis, POD patients had the same median blood pressure as control patients, but both absolute and sustained peak pressures were slightly elevated. They also spent longer times at low pressures up to 40 mmHg, both absolute and normalized. This suggests that longer exposure to malperfusion is a potential trigger of POD. However, none of the blood pressure-related parameters were retained in the final multivariable model. The inclusion of interaction terms between blood flow and blood pressure did not enhance the model fit, suggesting that the relationship between low flow and pressure does not significantly contribute to the predictive accuracy for POD. A meta-analysis reported no influence of high or low blood pressure targets on the incidence of POD [14], although a randomized trial demonstrated that exceeding the individual lower pressure limits of cerebral autoregulation is beneficial [15]. This leaves the extremes as potential triggers of POD.

POD patients had a significantly higher minimal blood pH and spent longer times at pH 7.2 and higher. The latter partially reflects their longer CPB times. Blood pH is related to the efficacy of gas exchange. Insufficient removal of carbon dioxide, e.g. during phases of high CPB blood flow, mandated by low systemic perfusion, potentially decreases blood pH. On the other hand, lower CPB blood flow could lead to an increased removal of carbon dioxide leading to a higher pH. During deep hypothermic interventions, such as those involving selective cerebral perfusion, acid–base management follows different principles compared to normothermic conditions. While our univariate analysis identified both deep cooling and selective cerebral perfusion as significant risk factors for POD, these variables did not reach significance in the minimal adequate model. Additionally, upon further examination, patient blood temperature was not significantly correlated with the incidence of POD. Although PaCO_2_ was not an independent predictor of POD, there was a lower minimal PaCO_2_ in POD patients compared to the controls. These patients also spent longer times at up to 40 mmHg, but lower fractions of total time at 40–60 mmHg of PaCO_2_. These data suggest an excellent gas exchange which may be related to efforts to improve oxygen saturation or blood pressure. However, blood vessels dilate, and blood pressure drops as blood pH decreases, further promoting systemic hypoperfusion. Kilgannon *et al.* [16] were able to show moderate hypercapnia between 50 and 70 mmHg as optimal for the neurological result in patients after cardiac arrest. This range coincides with our value intervals, in which no correlation for the development of POD was shown.

In the present study, POD patients did not differ in median, extreme and sustained extreme PaO_2_ values from non-POD patients. However, they spent lower fractions of total CPB time at PaO_2_ between 110 and 240 mmHg, but longer times and higher fractions of total time above 240 mmHg. This shift is a likely consequence of efforts to stabilize blood pressure by increased flow. The longest contiguous interval of PaO_2_ values between 110 and 140 mmHg was part of the final multivariable regression model but was not considered significant. The delirium-promoting influence of reduced oxygen partial pressure can be explained by hypoxia. Fujii *et al.* [17] were able to show increased oxidative stress for increased oxygen partial pressures during ECC. This resulted in an increase of pro-inflammatory cytokines IL-6 and TNF-α, as well as down-regulation of the anti-inflammatory IL-10, especially at pO2 values above 300 mmHg. Smulter *et al.* [18] investigated the role of oxygen saturation during CPB. Although there was no difference in mean venous oxygen saturation between POD patients and controls, the former spent a longer time at a saturation below 75%. This coincided with longer CPB times. A recent meta-analysis confirmed the utility of monitoring the cerebral oxygen saturation to prevent POD [19], whereas an earlier analysis did not find sufficient evidence [20].

Minimal invasive extracorporeal circulation (MiECC) was incorporated into the minimal adequate model as a protective factor against POD; however, it did not reach statistical significance in our study. The potential protective effects of MiECC could be related to its innovative design elements. These include a reduced priming volume that lessens hemodilution and the use of biocompatible coatings to minimize inflammatory responses. Additionally, its closed system design reduces the air-blood interface, which can decrease the risk of air embolism. The system also uses centrifugal pumps that are gentler on blood cells, along with integrated arterial filters to capture emboli. The reduced or absent venous reservoir further limits the blood's contact with artificial surfaces, potentially lowering the systemic inflammatory response. These features collectively could contribute to the theoretical benefits of MiECC in preventing postoperative complications like delirium. However, these findings must be cautiously interpreted as MiECC is often selected for less complex surgeries, which may introduce a bias in its perceived effectiveness against delirium. Similarly, Calafiore cardioplegia features in the minimal adequate model as a potential protective factor against POD, although it did not reach statistical significance; its presence may be related to generally shorter ischaemic times required for less complex procedures. In contrast, Bretschneider cardioplegia, which accommodates longer ischaemic durations, suggests a varied application in more extensive surgical contexts.

The underlying patho-mechanisms of delirium development are still not fully understood, although 2 mechanisms are often discussed as causal for POD: hypoxia and neuroinflammation [21]. Hypoperfusion, low blood pressure, deviations in pH and blood gases, all count towards hypoxic genesis. Hyperperfusion is most likely due to an inflammatory process in the sense of systemic inflammatory response syndrome.

Based on the insights gleaned from our study, we have refined our CPB protocol to achieve optimal perfusion. This involves a carefully calibrated increase in flow rate to mitigate the risk of hypoperfusion without inadvertently causing hyperperfusion. This change is meant to enhance cerebral perfusion within safe parameters, potentially reducing the incidence of POD. Simultaneously, we recognize that maintaining higher flow rates might necessitate acceptance of lower perfusion pressures at times. However, we remain vigilant to keep pressures above the lower limit of cerebral autoregulation, thus safeguarding against insufficient cerebral perfusion.

One of the purposes of the present study was to assess the utility of algorithm-based data processing to get further insight into the development of POD. This helped to pinpoint the influence of particular flow and oxygen partial pressure ranges on POD. Further refinements of this approach may provide additional insight and may help to define additional markers for the risk of developing POD.

### Limitations

The present study has some limitations that should be considered. First, CPB is just 1 piece of the POD puzzle. There are many more factors that contribute to POD. As this study focused on CPB as a risk factor for the incidence of POD, rather than duration or quality, only preoperative and intraoperative factors were considered, and postoperative influences such as drug therapy or ventilation strategy in the ICU were not evaluated. Additionally, the study did not examine the influence of anaesthesia during the procedure on the development of POD. Furthermore, the study's retrospective design implies that POD was not evaluated specifically for the study, potentially leading to non-recognition of hypoactive delirium forms. Nonetheless, the study's 14.4% incidence rate aligns with current delirium studies [2, 22]. The inclusion of the variable ‘year’ in our minimal adequate model revealed a slight uptick in the incidence of POD over time. This trend may reflect a heightened awareness and diagnostic vigilance but was certainly also subject to changes of staff and equipment and, above all, the increasing complexity of surgical procedures.

The retrospective design leads to an incomplete availability of data and causality must be interpreted with caution. One example is the lack of neuromonitoring data in this dataset. This is a limitation to this study because it could have given important insights on the influence of the CPB on cerebral perfusion. Nevertheless, this study’s aim was rather to determine which perfusion states are associated with POD and then to analyse the neurological parameters, which are influenced by perfusion itself. Furthermore, the study's sample size (3163 patients) and comprehensive CPB data granularity permit the detection of even minor factors that may impact the development of POD. Thus, these findings provide valuable insights for generating hypotheses for future prospective studies.

## CONCLUSION

This study reveals the substantial role of CPB parameters, specifically an increase in total CPB blood volume flow, blood flow extremes and deviations from the target value in the development of POD, underlining the need for careful management of CPB parameters in cardiac surgery. A comprehensive approach to CPB management requires understanding that these parameters cannot be monitored and regulated in isolation, because they are interrelated and influence each other. A closer examination of the operative factors and CPB parameters in connection with the simultaneous study of disturbances in cerebral perfusion, by routinely performing neuromonitoring would be important in the future. Furthermore, a more detailed examination of the blood gases in the arteries supplying the brain or the perioperative measurement of inflammatory markers should be considered to identify patho-mechanisms. Ultimately, a deeper understanding of these mechanisms will aid in the development of effective prevention strategies, thereby improving patient outcomes following cardiac surgery. The use of algorithm-based data processing, as demonstrated in this study, should be implemented routinely in future investigations to provide more comprehensive datasets, facilitating a deeper exploration of different CPB operational states on patient outcomes.

## Supplementary Material

ivae016_Supplementary_Data

## Data Availability

The data underlying this article will be shared on reasonable request to the corresponding author. **Johannes Krefting:** Conceptualization; Data curation; Formal analysis; Methodology; Project administration; Resources; Software; Supervision; Validation; Visualization; Writing—original draft; Writing—review & editing. **Hagen Gorki:** Conceptualization; Methodology; Supervision. **Markus Hoenicka:** Conceptualization; Data curation; Formal analysis; Methodology; Software; Supervision; Validation; Writing—original draft; Writing—review & editing. Günter Albrecht**:** Data curation; Resources. **Robert Kraft:** Data curation; Methodology; Resources; Software. **Andreas Liebold:** Project administration; Supervision; Writing—review & editing. Interdisciplinary CardioVascular and Thoracic Surgery thanks Carlos A. Mestres, Kerem M. Vural, Katrien Francois, Thomas Martens, Sven Martens and the other anonymous reviewer(s) for their contribution to the peer review process of this article.

## Notes

Presented at the 51st Annual Meeting of the Deutsche Gesellschaft für Thorax-, Herz- und Gefäßchirurgie (DGTHG) HERZMEDIZIN 2022, Hamburg, Germany, 18 February 2022.

## ABBREVIATIONS

ACC: Aortic cross-clamp
CI: Confidence interval
CPB: Cardiopulmonary bypass
ECC: Extracorporeal circulation
GLMs: Generalized linear models
POD: Postoperative delirium
