# Supplementary material for: Target flow deviations on the cardiopulmonary bypass cause postoperative delirium in cardiothoracic surgery—a retrospective study evaluating temporal fluctuations of perfusion data
Source: Interdiscip Cardiovasc Thorac Surg. 2024 Jan 30;38(2):ivae016. doi: 10.1093/icvts/ivae016 (PMC10853608; doi:10.1093/icvts/ivae016)
Supplement: ivae016_Supplementary_Data [file ivae016_supplementary_data.zip › Supplement.docx]

**Target flow deviations on the cardiopulmonary bypass cause postoperative delirium in cardiothoracic surgery – a retrospective study evaluating temporal fluctuations of perfusion data**

**Supplemental Material**

Johannes Krefting^1^; Hagen Gorki, MD^1^; Markus Hoenicka, PhD^1^; Günter Albrecht^1^; Robert Kraft, MD^1^; Andreas Liebold, MD^1^

^1^Department of Cardiothoracic and Vascular Surgery, Ulm University Medical Center, Ulm, Germany

Contents

[Contents 1](#_Toc155616865)

[Supplemental figure 1: Kernel density plots 2](#_Toc155616866)

[Supplemental figure 2: Receiver-operating characteristic analysis 3](#_Toc155616867)

[Supplemental table 1: Types of surgeries performed 4](#_Toc155616868)

[Supplemental table 2: Multiple regression analysis parameter definitions 5](#_Toc155616869)

[Supplemental table 3: Analysis of age and sex distribution 11](#_Toc155616870)

[Supplemental table 4: Perioperative data 12](#_Toc155616871)

[Supplemental table 5: Generalized linear regression models 13](#_Toc155616872)

[Supplemental table 6: Detailed univariable analysis of cardiopulmonary bypass implementation 15](#_Toc155616873)

[Supplemental table 7: Predictors of postoperative delirium as identified by multiple imputation and multivariable logistic regression. 26](#_Toc155616874)

[Supplemental table 8: Missingness of imputed variables 27](#_Toc155616875)

[Supplemental table 9: Combinations and Frequencies of CPB Equipment Utilization. 29](#_Toc155616876)

# Supplemental figure 1: Kernel density plots


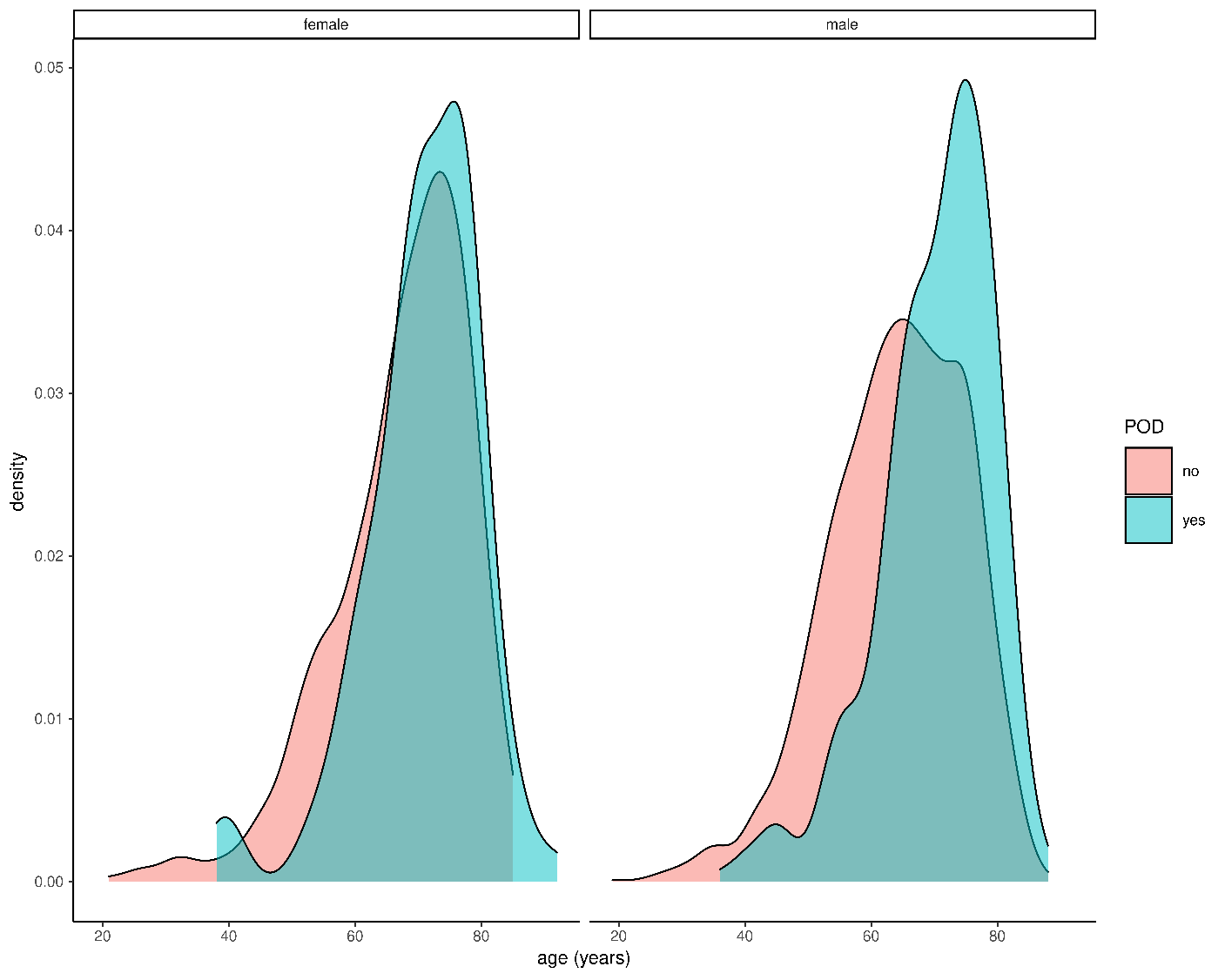


Supplemental figure 1: Kernel density plots of the age of male and female patients with and without postoperative delirium (POD). n=3163.

#
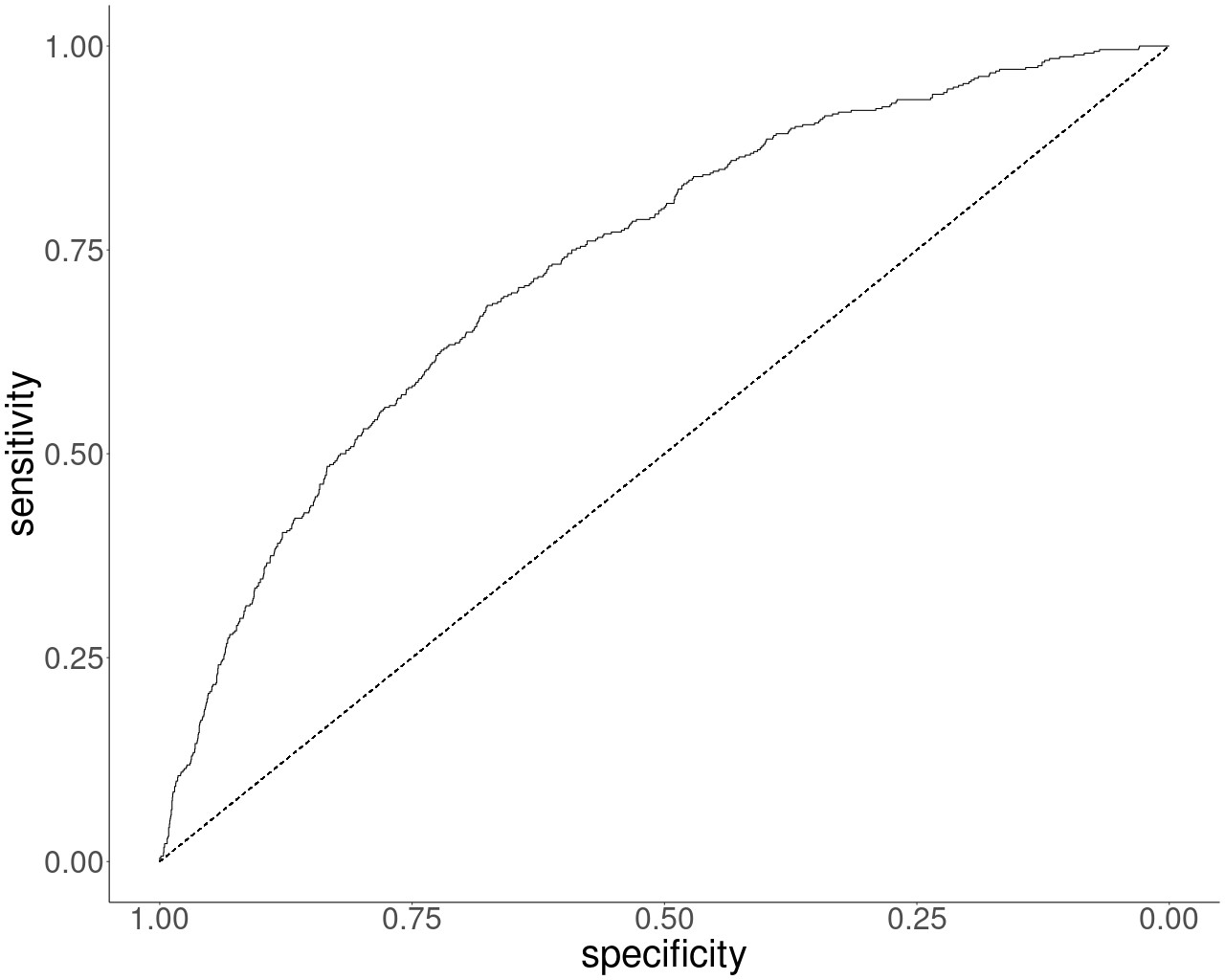
Supplemental figure 2: Receiver-operating characteristic analysis

Supplemental figure 2: Receiver-operating characteristic analysis of the risk of developing postoperative delirium using the final regression model and the pooled predictions of 10 imputations (n=3163). The dashed line indicates the line of no discrimination.

# Supplemental table 1: Types of surgeries performed

| Valve and aortic surgery | Mitral valve reconstruction/replacement  Aortic valve reconstruction/replacement  Tricuspidal valve reconstruction/replacement  David procedure  Bentall procedure  Ascending aorta replacement  Aortic bulbus reconstruction  Aortic (partial) arch replacement |
| --- | --- |
| Coronary Bypass surgery | All kinds of on pump aortocoronary bypasses under sternotomy |
| Other | Left ventricular assist device implantation  Left ventricular reduction  Myxoma/ cyst resection  Myectomy  Left atrial appendage-occlusion  Patent foramen ovale-occlusion |
| Minimal invasive surgery | Cardiacthoracic surgery where no (partial) sternotomy was performed:  Minimal-invasive bypass-operation  Minimal invasive surgery of the mitral- or tricuspic valve  Minimal invasive myxoma/ cyst resection |
| Combined surgery | Any combination consisting of at least two from the above |

# Supplemental table 2: Multiple regression analysis parameter definitions

| Parameter | Definition |
| --- | --- |
| postoperative delirium | Patient with documented postoperative delirium |
| male sex | Patient with male sex: yes/no |
| acute kidney failure | Occurrence of acute kidney failure in perioperative state: yes/no |
| diabetes mellitus | Presence of diabetes mellitus as comorbidity in patient: yes/no |
| right heart failure | Presence of right heart failure as comorbidity in patient: yes/no |
| atrial fibrillation | Presence of atrial fibrillation as comorbidity in patient: yes/no |
| history of cardiopulmonary resuscitation | History of cardiopulmonary resuscitation: yes/no |
| reservoir | Type of venous reservoir used in CPB system: Sorin Synthesis R, Maquet VHK 7100, Sorin D744,  Medos Reservoir, Euroset Admiral, Euroset Admicard 3200, LivaNova Inspire, Euroset Horizon, other |
| oxygenator | Type of oxygenator used in CPB system: Sorin Inspire, Terumo Capiox, Hilite 7000, Maquet Quadrox, Sorin Synthesis,  Paragon, Euroset Skipper, Medtronic Fusion, other |
| CPB system | Type of CPB system: conventional heart-lung machine (Stöckert models S3 and S5) vs. minimally invasive extracorporeal circulation: yes/no |
| vent location | Location of cardiac vent: Aorta, left ventricle, left atrium, right atrium, Vena pulmonalis, mobile cannula, other |
| cardioplegia set | Type of cardioplegia: Bretschneider cardioplegia solution vs. Calafiore cardioplegia |
| tub pack | Type of tubes used in CPB system |
| type of surgery | Type of surgery: aorto-coronary bypass surgery, singular aortic or valve surgery, combine surgery procedure, other |
| blood transfusion | Usage of blood transfusion: yes/no |
| emergency surgery | Non-elective surgery: yes/no |
| age on entry | Patient’s age at hospitalization |
| year of surgery | Year in which surgery was performed |
| BMI | Body mass index in kg·m^-2^ |
| BSA | Body surface area in m^2^ |
| target flow | Blood flow target value in L·min^-1^ |
| lowest CPB blood flow absolute | Lowest CPB blood flow absolute in L·min^-1^ |
| lowest CPB blood flow absolute windowed for 140 sec. | Lowest CPB blood flow in L·min^-1^ windowed for 140 sec. |
| lowest CPB blood flow relative | Lowest CPB blood flow absolute in % of target flow |
| lowest CPB blood flow relative windowed for 140 sec. | Lowest CPB blood flow in % of target flow windowed for 140 sec. |
| CBP blood flow abs. 0 incl. to 0.75 L·min^-1^·m^-2^ excl. total | Total time in seconds during CBP time for blood flow rate between 0 (included) and 0.75 L·min^-1^·m^-2^ (excluded) |
| CBP blood flow abs. 3.0 L·min^-1^·m^-2^ incl. to inf total | Total time in seconds during CBP time for blood flow rate from 3.0 L·min^-1^·m^-2^ (included) and higher |
| CBP blood flow abs. up to 0.75 L·min^-1^·m^-2^ excl. cont. | Total time in seconds during CBP time for blood flow rate of 6 L·min^-1^ (included) or higher |
| CBP blood flow abs. 3.0 L·min^-1^·m^-2^ incl. to inf cont | Longest continuous time in seconds during CBP time for blood flow rate of 6 L·min^-1^ (included) or higher |
| CBP blood flow deviation to target value min abs. | Lowest negative blood flow deviation to target value in % |
| CBP blood flow deviation to target value min windowed 140 sec. | Lowest negative blood flow deviation to target value in % windowed for 140 sec. |
| CBP blood flow deviation to target value minus inf incl. to minus 50 % excl. total | Total time in seconds during CBP time for CBP blood flow deviation to target value from minus infinite (excluded) to minus 50 % (excluded) |
| CBP blood flow deviation to target value minus inf incl. to minus 50 % excl. cont. | Longest continuous time in seconds during CBP time for blood flow deviation to target value from minus infinite (excluded) to minus 50 % (excluded) |
| CBP blood flow deviation to target value 10% incl. to 30% excl. total | Total time in seconds during CBP time for CBP blood flow deviation to target value between 10% (included) and 30% (excluded) |
| CBP blood flow deviation to target value 30% incl. to inf excl. total | Total time in seconds during CBP time for CBP blood flow deviation to target value of 30% (included) or higher |
| CBP blood flow deviation to target value 30% incl. to inf excl. cont. | Longest continuous time in seconds during CBP time for blood flow deviation to target value of 30% (included) or higher |
| patient blood pressure minus inf incl. to 40 mmHg excl. total | Total time in seconds during CBP time for patient blood pressure between 0 (included) and 40 mmHg (excluded) |
| patient blood pressure 40 incl. to 60 mmHg excl. total | Total time in seconds during CBP time for patient between 40 (included) and 60 mmHg (excluded) |
| pH art. min abs. | Lowest arterial blood pH absolute |
| pH art. 7.2 incl. to 7.4 excl. total | Total time in seconds during CBP time for arterial blood pH between 7.2 (included) and 7.4 (excluded) |
| pH art. 7.6 incl. to inf excl. total | Total time in seconds during CBP time for pH art. 7.6 (included) to inf (excluded) |
| pH art. 7.6 incl. to inf excl. cont. | Longest continuous time in seconds during CBP time for arterial blood pH of 7.6 (included) or higher |
| pCO2 art. minus inf incl. to 20 mmHg excl. total | Total time in seconds during CBP time for arterial blood pCO2 between 0 (included) and 20 mmHg (excluded) |
| pCO2 art. 20 incl. to 30 mmHg excl. total | Total time in seconds during CBP time for arterial blood pCO2 between 20 (included) and 30 mmHg (excluded) |
| pCO2 art. 20 incl. to 30 mmHg excl. cont. | Longest continuous time in seconds during CPB time for arterial blood pCO2 between 20 (included) and 30 mmHg (excluded) |
| pCO2 art. 30 incl. to 40 mmHg excl. total | Total time in seconds during CBP time for arterial blood pCO2 between 30 (included) and 40 mmHg (excluded) |
| pCO2 art. 30 incl. to 40 mmHg excl. cont. | Longest continuous time in seconds during CPB time for arterial blood pCO2 between 30 (included) and 40 mmHg (excluded) |
| pO2 art. minus inf incl. to 70 mmHg excl. cont. | Longest continuous time in seconds during CPB time for arterial blood pO2 between 0 (included) and 70 mmHg (excluded) |
| pO2 art. 110 incl. to 140 mmHg excl. total | Total time in seconds during CBP time for arterial blood pO2 between 110 (included) to 140 mmHg (excluded) |
| pO2 art. 110 incl. to 140 mmHg excl. cont. | Longest continuous time in seconds during CPB time for arterial blood pO2 between 110 (included) and 140 mmHg (excluded) |
| pO2 art. 240 mmHg incl. to inf excl. total | Total time in seconds during CBP time for arterial blood pO2 of 240 mmHg (included) or higher |
| pO2 art. 240 mmHg incl. to inf excl. cont. | Longest continuous time in seconds during CPB time for arterial blood pO2 of 240 mmHg (included) or higher |
| temperature minus inf incl. to 31°C excl. total | Total time in seconds during CBP time for temperature minus inf (included) to 31°C (excluded) |
| temperature 31°C incl. to 33°C excl. total | Total time in seconds during CBP time for temperature 31°C (included) to 33°C (excluded) |
| temperature 31°C incl. to 33°C excl. cont. | Longest continuous time in seconds during CPB time for temperature 31°C (included) to 33°C (excluded) |
| minimal Hct – % | Lowest hematocrit in % absolute |
| maximal Hct – % | Highest hematocrit in % absolute |
| minimal Hct [140 s] – % | Lowest hematocrit in % windowed for 140 sec. |
| maximal Hct [140 s] – % | Highest hematocrit in % windowed for 140 sec. |
| Hct [up to 20%[ – s | Total time in seconds during CBP time for hematocrit below 20% |
| Hct [20% to 30%[ – s | Total time in seconds during CBP time for hematocrit from 20% to 30% |
| Hct [30% to 40%[ – s | Total time in seconds during CBP time for hematocrit from 30% to 40% |
| ‍Hct [40% and more] – s | Total time in seconds during CBP time for hematocrit above 40% |
| ‍cont. Hct [up to 20%[ – s | Longest continuous time in seconds during CPB time for hematocrit below 20% |
| cont. Hct [20% to 30%[ – s | Longest continuous time in seconds during CPB time for hematocrit from 20% to 30% |
| cont. Hct [30% to 40%[ – s | Longest continuous time in seconds during CPB time for hematocrit from 30% to 40% |
| ‍cont. Hct [40% and more] – s | Longest continuous time in seconds during CPB time for hematocrit above 40% |
| total CBP blood volume | Total CBP blood volume in liters |
| total CBP time | Total CBP time in minutes |
| aortic cross-clamp time | Aortic cross-clamp time in minutes |
| reperfusion time | Reperfusiontime in minutes |
| lowest total temperature | Lowest blood temperature of patient during CPB in °C |
| deep cooling | Intentionally lowering the body temperature to reduce metabolic needs and protect the organs |
| selective brain perfusion | Maintaining blood flow, selectively to the brain, during operations on the aortic arch |

CPB = cardiopulmonary bypass

Supplemental table 3: Analysis of age and sex distribution

|  | Controls – no delirium | Cases – delirium | Total | Difference in age  Adjusted p (Holm) |
| --- | --- | --- | --- | --- |
| Female  Age (years)  n | 69 (62 to 75)  696 | 72 (66 to 77)  97 | 70 (62 to 75)  793 (12% delirium) | 0.009 |
| Male  Age (years)  n | 65 (57 to 72)  2011 | 72 (65 to 77)  359 | 66 (58 to 73)  2370 (15% delirium) | <0.0001 |
| Total  Age (years)  n | 66 (58 to 73)  2707 (74% male) | 72 (66 to 77)  456 (79% male) | 67 (59 to 74)  3163 (14% delirium, 75% male) |  |
| Difference in age  Adjusted p (Holm) | <0.0001 | 0.62 |  |  |

Age data are presented as median (1st quartile to 3rd quartile). POD, postoperative delirium. Analysis of variance indicated a significant influence of sex (p<0.0001) and age (p<0.0001) on POD with a significant interaction between the factors (p=0.04). Patients with POD were older than control patients irrespective of their sex. The age of male and female POD patients was not significantly different, whereas male control patients were significantly younger than female control patients.

# Supplemental table 4: Perioperative data

|  | controls – no delirium  (n=2707) | cases – delirium  (n=456) | total  (n=3163) | statistic | p |
| --- | --- | --- | --- | --- | --- |
| type of surgery – no. (%)  aortocoronary bypass  isolated valve or aortic surgery  combined surgery  minimally invasive cardiac surgery  other | 1225 (45.2%)  449 (16.6%)  732 (27.0%)  244 (9.0%)  57 (2.1%) | 174 (38.2%)  80 (17.5%)  173 (37.9%)  25 (5.5%)  4 (0.8%) | 1399 (44.2%)  529 (16.7%)  905 (28.6%)  269 (8.5%)  61 (1.9%) | (reference)  OR: 1.26 (CI: 0.94 to 1.67)  OR: 1.66 (CI: 1.32 to 2.09)  OR: 0.73 (CI: 0.46 to 1.11)  OR: 0.51 (CI: 0.15 to 1.27) | 0.12  <0.0001  0.14  0.16 |
| urgency – no. (%)  elective  urgent  emergency | 2231 (82.4%)  79 (2.9%)  396 (14.6%) | 345 (75.7%)  24 (5.2%)  87 (19.1%) | 2576 (81.5%)  103 (3.2%)  483 (15.3%) | (reference)  OR: 1.97 (CI: 1.21 to 3.11)  OR: 1.42 (CI: 1.09 to 1.83) | 0.008  0.009 |
| cardiopulmonary bypass time – min | 113.7 (92.2 to 143.0) | 133.9 (104.6 to 170.3) | 116.1 (93.5 to 147.1) | r_pb_:0.13 (CI: 0.09 to 0.16) | <0.0001 |
| cross-clamp time – min | 66.5 (51.9 to 86.7) | 77.9 (58.5 to 104.4) | 67.9 (52.6 to 89.5) | r_pb_:0.13 (CI: 0.09 to 0.16) | <0.0001 |
| reperfusion time – min | 32.5 (24.9 to 41.9) | 38.0 (28.9 to 48.4) | 33.2 (25.3 to 43.0) | r_pb_:0.11 (CI: 0.07 to 0.14) | <0.0001 |
| target flow – L·min^-1^ | 4.70 (4.37 to 5.04) | 4.67 (4.37 to 4.97) | 4.70 (4.37 to 5.02) | r_pb_:-0.04 (CI: -0.07 to -0.004) | 0.029 |
| total cardiopulmonary bypass flow - L | 521 (415 to 656) | 602 (473 to 787) | 531 (421 to 672) | r_pb_: 0.14 (CI: 0.11 to 0.18) | <0.0001 |

Continuous numeric data are presented as mean (1^st^ quartile to 3^rd^ quartile). OR, unadjusted odds ratio; r_pb_, point-biserial correlation coefficient; CI, 95% confidence interval.

# Supplemental table 5: Generalized linear regression models

| model type | model formula |
| --- | --- |
| maximal model | postoperative delirium ~  age on entry + male sex + acute kidney failure + chronic kidney failure + carotid disease + arterial hypertension + hyperlipoproteinemia + atrial fibrillation + diabetes mellitus + right heart failure + history of cardiopulmonary resuscitation + emergency surgery + year of surgery + *BMI + BSA* + oxygenator + reservoir + CPB system + vent location+ cardioplegia set + tub pack + type of surgery + deep cooling + selective cerebral perfusion + blood transfusion + target flow  + *lowest CPB blood flow absolute*  + *lowest CPB blood flow absolute windowed for 140 sec.*  + *lowest CPB blood flow relative*  + lowest CPB blood flow relative windowed for 140 sec.  + CBP blood flow abs. 0 incl. to 0.75 L·min^-1^·m^-2^excl. total  + *CBP blood flow abs. 0 incl. to 0.75 L·min^-1^·m^-2^ excl. cont.*  + *CBP blood flow abs. 3.0 incl. to 3.75 L·min^-1^·m^-2^ excl. total*  + CBP blood flow abs. 3.75 L·min^-1^·m^-2^ incl. to inf excl. total  + *CBP blood flow abs. 3.75 L·min^-1^·m^-2^ incl. to inf excl. cont.*  + *CBP blood flow deviation from target value minus inf incl. to minus 50 % excl. total*  + CBP blood flow deviation from target value minus inf incl. to minus 50 % excl. cont.  *+ CBP blood flow deviation from target value 10% incl. to 30% excl. total*  + CBP blood flow deviation from target value 10% incl. to 30% excl. cont  *+ CBP blood flow deviation from target value 30% incl. to inf excl. total*  + CBP blood flow deviation from target value 30% incl. to inf excl. cont.  + patient blood pressure minus inf incl. to 40 mmHg excl. total  + patient blood pressure 40 incl. to 60 mmHg excl. total  + pH art. 7.2 incl. to 7.4 excl. total  + pH art. 7.6 incl. to inf excl. total  *+ pH art. 7.6 incl. to inf excl. cont.*  + pCO2 art. 0 incl. to 20 mmHg excl. total  + pCO2 art. 20 incl. to 30 mmHg excl. total  + pCO2 art. 20 incl. to 30 mmHg excl. cont.  + pCO2 art. 30 incl. to 40 mmHg excl. total  + pCO2 art. 30 incl. to 40 mmHg excl. cont.  *+ pO2 art. 0 incl. to 70 mmHg excl. cont.*  *+ pO2 art. 110 incl. to 140 mmHg excl. total*  + pO2 art. 110 incl. to 140 mmHg excl. cont.  + pO2 art. 240 mmHg incl. to inf excl. total  *+ pO2 art. 240 mmHg incl. to inf excl. cont.*  + Hct art. 20% incl. to 30% excl. total  *+ Hct art. 20% incl. to 30% excl. cont*  + Hct art. 30% incl. to 40% excl. total  *+ Hct art. 30% incl. to 40% excl. cont*  + temperature minus inf incl. to 31 excl. total  + temperature 31°C incl. to 33°C excl. total  *+ temperature 31°C incl. to 33°C excl. cont.*  + total CBP blood volume *+ total CBP time* + aortic cross-clamp time + reperfusion time + lowest total temperature |
| intermediate consensusminimal adequate model | postoperative delirium ~  male sex + acute kidney failure + carotid disease + chronic renal failure + diabetes mellitus + CPB system + cardioplegia set + emergency surgery + age on entry + year of surgery + target flow + lowest CPB blood flow relative windowed for 140 sec. + CBP blood flow deviation to target value 30% incl. to inf excl. cont. + pO2 art. 110 incl. to 140 mmHg excl. cont. + Hct 30 incl. to 40% excl. total + total CPB blood volume |

Factors were considered for inclusion into the maximal model according to the results of the univariate analysis. Factors in *italics* were excluded from the maximal model owing to correlations of 0.8 or higher with other factors which remained in the model. Ten imputations were calculated and evaluated with GLMs independently. All imputations resulted in the same step-wise simplified model. This model also resulted in the minimum of the arithmetic mean of the Akaike Information Criterion of the ten imputations and thus constituted the minimal adequate model. CPB = cardiopulmonary bypass; BMI = body mass index; BSA = body surface area; incl. = included; excl. = excluded; cont. = continuous; abs. = absolute

# Supplemental table 6: Detailed univariable analysis of cardiopulmonary bypass implementation

|  | controls – no delirium  (n=2707) | delirium  (n=456) | total  (n=3163) | statistic | p |
| --- | --- | --- | --- | --- | --- |
| **I. Flow**  **I.1. Absolute flow** |  |  |  |  |  |
| median flow – L·min^-1^  - L·min^-1^·m^-2^ | 4.66 (4.27 to 5.03)  2.39 (2.20 to 2.51) | 4.63 (4.28 to 5.03  2.40 (2.24 to 2.55) | 4.64 (4.27 to 5.03)  2.39 (2.21 to 2.51) | r_pb_:-0.002 (-0.037 to 0.033) | 0.90 |
| minimal flow – L·min^-1^  - L·min^-1^·m^-2^ | 1.03 (0.22 to 1.59)  0.523 (0.114 to 0.811) | 0.72 (0.13 to 1.38)  0.378 (0.061 to 0.717) | 1.02 (0.20 to 1.56)  0.502 (0.104 to 0.799) | r_pb_:-0.07 (-0.11 to -0.04) | **<0.0001** |
| maximal flow – L·min^-1^  - L·min^-1^·m^-2^ | 5.52 (5.12 to 5.96)  2.79 (2.62 to 3.03) | 5.57 (5.12 to 6.00)  2.85 (2.65 to 3.11) | 5.52 (5.12 to 5.96)  2.80 (2.62 to 3.04) | r_pb_:0.03 (-0.01 to 0.06) | 0.17 |
| minimal flow [140 s] – L·min^-1^  - L·min^-1^·m^-2^ | 2.98 (2.09 to 3.54)  1.54 (1.09 to 1.81) | 2.56 (1.60 to 3.27)  1.33 (0.82 to 1.72) | 2.92 (2.00 to 3.51)  1.51 (1.05 to 1.80) | r_pb_: -0.12 (-0.15 to -0.08) | **<0.0001** |
| maximal flow [140 s] - L·min^-1^  - L·min^-1^·m^-2^ | 5.43 (5.06 to 5.81)  2.74 (2.56 to 2.95) | 5.47 (5.03 to 5.90)  2.79 (2.62 to 3.03) | 5.43 (5.05 to 5.82)  2.75 (2.57 to 2.97) | r_pb_: 0.035 (0.00 to 0.07) | **0.049** |
| **I.2. Cumulative time within BSA normalized flow rate range** |  |  |  |  |  |
| total flow [up to 0.75 L·min^-1^·m^-2^ [ - s  - % of total time | 20 (0 to 40)  0.23 (0 to 0.57) | 20 (0 to 60)  0.28 (0 to 0.91) | 20 (0 to 40)  0.24 (0 to 0.60) | r_pb_: 0.07 (0.04 to 0.10)  r_pb_: 0.08 (0.04 to 0.11) | **<0.0001**  **<0.0001** |
| total flow [0.75 to 1.5 L·min^-1^·m^-2^ [ - s  - % of total time | 60 (20 to 140)  0.91 (0.41 to 1.91) | 100 (40 to 220)  1.13 (0.55 to 2.44) | 60 (20 to 140)  0.94 (0.43 to 1.96) | r_pb_: 0.06 (0.02 to 0.09)  r_pb_: 0.02 (-0.01 to 0.06) | **0.0017**  0.242 |
| total flow [1.5 to 2.25 L·min^-1^·m^-2^ [ - s  - % of total time | 1520 (380 to 3400)  22.40 (5.98 to 50.00) | 1560 (400 to 3700)  18.35 (5.44 to 44.19) | 1520 (400 to 3440)  21.68 (5.90 to 49.42) | r_pb_: 0.02 (-0.02 to 0.05)  r_pb_: -0.03 (-0.07 to 0.00) | 0.265  0.068 |
| total flow [2.25 to 3.0 L·min^-1^·m^-2^ [ - s  - % of total time | 4180 (2610 to 6000)  65.47 (41.23 to 85.60) | 4760 (3260 to 6870)  64.44 (43.04 to 83.38) | 4280 (2660 to 6140)  65.27 (41.51 to 85.41) | r_pb_: 0.08 (0.05 to 0.12)  r_pb_: -0.01 (-0.05 to 0.02) | **<0.0001**  0.473 |
| total flow [3.0 L·min^-1^·m^-2^ and more] - s  - % of total time | 0 (0 to 40)  0 (0 to 0.47) | 0 (0 to 190)  0 (0 to 2.1) | 0 (0 to 40)  0 (0 to 0.65) | r_pb_: 0.08 (0.05 to 0.12)  r_pb_: 0.05 (0.02 to 0.09) | **<0.0001**  **0.0034** |
| **I.3. Longest contiguous time interval within BSA normalized flow rate range** |  |  |  |  |  |
| cont. flow [up to 0.75 L·min^-1^·m^-2^[ - s  - % of total time | 20 (0 to 20)  0.20 (0 to 0.35) | 20 (0 to 20)  0.23 (0 to 0.41) | 20 (0 to 20)  0.20 (0 to 0.35) | r_pb_: 0.07 (0.03 to 0.10)  r_pb_: 0.07 (0.04 to 0.11) | **0.0001**  **<0.0001** |
| cont. flow [0.75 to 1.5 L·min^-1^·m^-2^[ - s  - % of total time | 20 (20 to 80)  0.44 (0.27 to 1.01) | 40 (20 to 120)  0.57 (0.31 to 1.43) | 20 (20 to 80)  0.45 (0.28 to 1.09) | r_pb_: 0.05 (0.02 to 0.09)  r_pb_: 0.02 (-0.01 to 0.06) | **0.0024**  0.21 |
| cont. flow [1.5 to 2.25 L·min^-1^·m^-2^[ - s  - % of total time | 760 (180 to 1780)  11.21 (2.92 to 26.19) | 680 (180 to 1720)  8.82 (2.44 to 22.29) | 760 (180 to 1780)  10.92 (2.84 to 25.29) | r_pb_: -0.00 (-0.03 to 0.04)  r_pb_: -0.04 (-0.08 to -0.01) | 0.95  **0.014** |
| cont. flow [2.25 to 3.0 L·min^-1^·m^-2^[ - s  - % of total time | 1980 (1160 to 3340)  29.05 (17.41 to 48.61) | 2040 (1180 to 3710)  26.42 (14.55 to 45.58) | 1980 (1160 to 3380)  28.67 (16.80 to 48.22) | r_pb_: 0.04 (0.00 to 0.07)  r_pb_: -0.04 (-0.07 to -0.002) | **0.034**  **0.040** |
| cont. flow [3.0 to 3.75 L·min^-1^·m^-2^[ – s  - % of total time | 0 (0 to 20)  0 (0 to 0.40) | 0 (0 to 150)  0 (0 to 1.84) | 0 (0 to 40)  0 (0 to 0.55) | r_pb_: 0.08 (0.04 to 0.11)  r_pb_: 0.05 (0.01 to 0.08) | **<0.0001**  **0.010** |
| cont. flow [3.75 L·min^-1^·m^-2^ and more] – s  - % of total time | 0 (0 to 0)  0 (0 to 0) | 0 (0 to 0)  0 (0 to 0) | 0 (0 to 0)  0 (0 to 0) | r_pb_: 0.06 (0.02 to 0.09)  r_pb_: 0.03 (-0.00 to 0.07) | **0.0011**  0.066 |
| **I.4. Deviation from target flow** |  |  |  |  |  |
| deviation of median flow - % of target flow | -0.41 (-8.13 to 4.54) | 0 (-6.59 to 6.10) | -0.39 (-7.89 to 4.71) | r_pb_: 0.02 (-0.01 to 0.06) | 0.23 |
| maximal negative flow deviation - % of target flow | -78.3 (-95.3 to 66.2) | -84.4 (-97.5 to -70.1) | -79.1 (-95.7 to -66.7) | r_pb_: -0.07 (-0.10 to -0.03) | **0.0002** |
| maximal positive flow deviation - % of target flow | 16.4 (9.1 to 26.1) | 18.8 (10.5 to 29.4) | 16.7 (9.3 to 26.9) | r_pb_: 0.04 (0.00 to 0.07) | **0.025** |
| maximal negative flow deviation [140 s] - % of target flow | -35.4 (-54.0 to -24.3) | -43.9 (-65.3 to -28.0) | -36.5 (-55.6 to -24.8) | r_pb_: -0.10 (-0.14 to -0.07) | **<0.0001** |
| maximal positive flow deviation [140 s] - % of target flow | 14.0 (6.7 to 22.9) | 16.1 (8.8 to 26.0) | 14.3 (6.8 to 23.5) | r_pb_: 0.05 (0.01 to 0.08) | **0.007** |
| **I.5 Cumulative time within flow deviation range** |  |  |  |  |  |
| total flow deviation [more than -50%[ - s  - % of total time | 80 (40 to 160)  1.2 (0.6 to 2.3) | 120 (60 to 280)  1.5 (0.8 to 3.3) | 80 (40 to 180)  1.2 (0.7 to 2.4) | r_pb_: 0.07 (0.04 to 0.11)  r_pb_: 0.07 (0.04 to 0.10) | **<0.0001**  **<0.0001** |
| total flow deviation [-50 to -30%[ - s  - % of total time | 80 (40 to 160)  1.1 (0.6 to 2.3) | 100 (40 to 220)  1.2 (0.6 to 2.5) | 80 (40 to 180)  1.1 (0.6 to 2.3) | r_pb_: 0.03 (0.00 to 0.07)  r_pb_: 0.00 (-0.03 to 0.04) | **0.049**  0.97 |
| total flow deviation [-30 to -10%[ - s  - % of total time | 760 (160 to 2140)  11.2 (2.4 to 31.1) | 780 (180 to 2400)  9.2 (2.2 to 28.9) | 760 (160 to 2180)  10.9 (2.3 to 30.6) | r_pb_: 0.02 (-0.02 to 0.05)  r_pb_: -0.03 (-0.06 to 0.00) | 0.29  0.091 |
| total flow deviation [-10 to 10%[ - s  - % of total time | 3700 (2240 to 5440)  56.1 (35.5 to 76.5) | 4010 (2265 to 6170)  51.6 (30.8 to 73.9) | 3780 (2240 to 5580)  55.6 (34.8 to 76.3) | r_pb_: 0.05 (0.02 to 0.09)  r_pb_: -0.05 (-0.08 to -0.01) | **0.0043**  **0.0070** |
| total flow deviation [10 to 30%[ - s  - % of total time | 580 (0 to 1880)  8.7 (0 to 26.6) | 980 (20 to 2450)  11.6 (0.3 to 31.1) | 640 (0 to 1960)  9.1 (0 to 26.9) | r_pb_: 0.07 (0.04 to 0.11)  r_pb_: 0.04 (0.00 to 0.07) | **<0.0001**  **0.040** |
| total flow deviation [30% and more] – s  - % of total time | 0 (0 to 0)  0 (0 to 0) | 0 (0 to 0)  0 (0 to 0) | 0 (0 to 0)  0 (0 to 0) | r_pb_: 0.08 (0.05 to 0.12)  r_pb_: 0.05 (0.01 to 0.08) | **<0.0001**  **0.0051** |
| **I.6 Longest contiguous time intervals within flow deviation range** |  |  |  |  |  |
| cont. flow deviation [more than -50%[ - s  - % of total time | 40 (20 to 100)  0.5 (0.3 to 1.3) | 40 (20 to 165)  0.7 (0.3 to 2.2) | 40 (20 to 100)  0.6 (0.3 to 1.4) | r_pb_: 0.07 (0.04 to 0.11)  r_pb_: 0.07 (0.03 to 0.10) | **<0.0001**  **0.0002** |
| cont. flow deviation [-50 to -30%[ - s  - % of total time | 40 (20 to 80)  0.6 (0.3 to 1.3) | 60 (20 to 120)  0.7 (0.4 to 1.4) | 40 (20 to 100)  0.6 (0.3 to 1.3) | r_pb_: 0.03 (-0.01 to 0.06)  r_pb_: -0.00 (-0.04 to 0.03) | 0.13  0.98 |
| cont. flow deviation [-30 to -10%[ - s  - % of total time | 380 (80 to 1060)  5.2 (1.2 to 15.6) | 350 (80 to 1140)  4.2 (1.1 to 13.5) | 360 (80 to 1080)  5.1 (1.2 to 15.3) | r_pb_: 0.01 (-0.02 to 0.05)  r_pb_: -0.03 (-0.07 to 0.00) | 0.48  0.052 |
| cont. flow deviation [-10 to 10%[ - s  - % of total time | 1660 (920 to 3000)  24.8 (13.8 to 42.9) | 1680 (920 to 3095)  20.2 (11.5 to 37.7) | 1660 (920 to 3000)  24.2 (13.3 to 42.1) | r_pb_: 0.01 (-0.03 to 0.04)  r_pb_: -0.06 (-0.10 to -0.03) | 0.63  **0.0003** |
| cont. flow deviation [10 to 30%[ - s  - % of total time | 360 (0 to 1040)  5.1 (0 to 14.2) | 560 (20 to 1300)  6.4 (0.3 to 15.4) | 380 (0 to 1060)  5.3 (0 to 14.5) | r_pb_: 0.07 (0.03 to 0.10)  r_pb_: 0.03 (-0.00 to 0.07) | **0.0001**  0.089 |
| cont. flow deviation [30% and more] – s  - % of total time | 0 (0 to 0)  0 (0 to 0) | 0 (0 to 0)  0 (0 to 0) | 0 (0 to 0)  0 (0 to 0) | r_pb_: 0.09 (0.05 to 0.12)  r_pb_: 0.05 (0.01 to 0.08) | **<0.0001**  **0.0053** |
| **II. Blood pressure**  **II.1. Absolute pressure** |  |  |  |  |  |
| median pressure – mmHg | 52 (48 to 58) | 52 (48 to 57) | 52 (48 to 58) | r_pb_: -0.03 (-0.06 to 0.01) | 0.13 |
| minimal pressure – mmHg | 27 (22 to 32) | 26 (21 to 32) | 27 (22 to 32) | r_pb_: -0.03 (-0.07 to 0.00) | 0.070 |
| maximal pressure – mmHg | 82 (75 to 92) | 83 (75.3 to 94) | 82 (75 to 92) | r_pb_: 0.06 (0.03 to 0.10) | **0.0007** |
| minimal pressure [140 s] – mmHg | 36 (32 to 40) | 36 (31 to 39) | 36 (32 to 40) | r_pb_: -0.04 (-0.08 to -0.01) | **0.020** |
| maximal pressure [140 s] - mmHg | 74 (67 to 82) | 76 (69 to 84) | 74 (67 to 82) | r_pb_: 0.07 (0.03 to 0.11) | **0.0001** |
| **II.2. Cumulative time within pressure range** |  |  |  |  |  |
| total pressure [up to 40 mmHg[ - s  - % of total time | 360 (140 to 840)  5.5 (2.3 to 12.1) | 480 (180 to 1080)  6.1 (2.5 to 13.8) | 380 (160 to 880)  5.6 (2.3 to 12.3) | r_pb_: 0.06 (0.03 to 0.10)  r_pb_: 0.04 (0.00 to 0.07) | **0.0004**  **0.044** |
| total pressure [40 to 60 mmHg[ - s  - % of total time | 4300 (2960 to 5720)  65.4 (48.4 to 78.4) | 4900 (3475 to 6645)  65.1 (48.3 to 78.1) | 4400 (3020 to 5860)  65.3 (48.4 to 78.4) | r_pb_: 0.11 (0.07 to 0.14)  r_pb_: 0.01 (-0.02 to 0.05) | **<0.0001**  0.55 |
| total pressure [60 to 80 mmHg[ - s  - % of total time | 1420 (540 to 2760)  21.2 (8.6 to 39.4) | 1590 (540 to 3080)  20.2 (7.3 to 37.1) | 1440 (540 to 2820  21.1 (8.5 to 39.1) | r_pb_: 0.04 (0.00 to 0.07)  r_pb_: -0.03 (-0.07 to 0.00) | **0.047**  0.078 |
| total pressure [80 to 100 mmHg[ - s  - % of total time | 20 (0 to 120)  0.3 (0 to 1.9) | 30 (0 to 160)  0.4 (0 to 1.9) | 20 (0 to 140)  0.3 (0 to 1.9) | r_pb_: 0.01 (-0.02 to 0.05)  r_pb_: -0.01 (-0.05 to 0.02) | 0.48  0.54 |
| total pressure [100 mmHg and more] - s  - % of total time | 0 (0 to 0)  0 (0 to 0) | 0 (0 to 0)  0 (0 to 0) | 0 (0 to 0)  0 (0 to 0) | r_pb_: 0.01 (-0.02 to 0.05)  r_pb_: 0.01 (-0.03 to 0.04) | 0.41  0.58 |
| **II.3. Longest contiguous time interval within pressure range** |  |  |  |  |  |
| cont. pressure [up to 40 mmHg[ - s  - % of total time | 120 (60 to 220)  1.7 (0.9 to 3.2) | 120 (60 to 280)  1.7 (0.9 to 3.4) | 120 (60 to 220)  1.7 (0.9 to 3.2) | r_pb_: 0.06 (0.03 to 0.09)  r_pb_: 0.03 (-0.00 to 0.07) | **0.0007**  0.064 |
| cont. pressure [40 to 60 mmHg[ - s  - % of total time | 1080 (720 to 1670)  16.2 (10.3 to 24.1) | 1230 (760 to 1825)  15.2 (9.9 to 23.3) | 1100 (720 to 1700)  16.1 (10.2 to 24.1) | r_pb_: 0.05 (0.02 to 0.09)  r_pb_: -0.01 (-0.05 to 0.02) | **0.0026**  0.47 |
| cont. pressure [60 to 80 mmHg[ - s  - % of total time | 400 (160 to 750)  5.8 (2.5 to 10.7) | 420 (180 to 760)  5.1 (2.4 to 9.3) | 400 (170 to 760)  5.6 (2.5 to 10.6) | r_pb_: 0.00 (-0.03 to 0.04)  r_pb_: -0.05 (-0.08 to -0.01) | 0.79  **0.0056** |
| cont. pressure [80 to 100 mmHg[ - s  - % of total time | 20 (0 to 80)  0.3 (0 to 1.1) | 20 (0 to 80)  0.3 (0 to 1.1) | 20 (0 to 80)  0.3 (0 to 1.1) | r_pb_: 0.01 (-0.03 to 0.04)  r_pb_: -0.02 (-0.06 to 0.01) | 0.62  0.25 |
| cont. pressure [100 mmHg and more] - s  - % of total time | 0 (0 to 0)  0 (0 to 0) | 0 (0 to 0)  0 (0 to 0) | 0 (0 to 0)  0 (0 to 0) | r_pb_: 0.02 (-0.01 to 0.06)  r_pb_: 0.02 (-0.02 to 0.05) | 0.19  0.34 |
| **III. Blood pH**  **III.1. Absolute pH** |  |  |  |  |  |
| median pH | 7.39 (7.36 to 7.41) | 7.39 (7.36 to 7.42) | 7.39 (7.36 to 7.42) | r_pb_: 0.01 (-0.02 to 0.05) | 0.47 |
| minimal pH | 7.21 (6.98 to 7.32) | 7.22 (7.04 to 7.32) | 7.22 (7.00 to 7.32) | r_pb_: 0.04 (0.00 to 0.09) | **0.046** |
| maximal pH | 7.51 (7.46 to 7.59) | 7.52 (7.47 to 7.61) | 7.51 (7.46 to 7.59) | r_pb_: 0.04 (-0.01 to 0.08) | 0.099 |
| minimal pH [140 s] | 7.24 (7.05 to 7.32) | 7.24 (7.05 to 7.31) | 7.24 (7.05 to 7.32) | r_pb_: -0.01 (-0.05 to 0.02) | 0.55 |
| maximal pH [140 s] | 7.49 (7.44 to 7.56) | 7.49 (7.45 to 7.56) | 7.49 (7.44 to 7.56) | r_pb_: 0.02 (-0.03 to 0.06) | 0.48 |
| **III.2. Cumulative time within pH range** |  |  |  |  |  |
| total pH [up to 6.8[ - s  - % of total time | 0 (0 to 20)  0 (0 to 0.4) | 0 (0 to 20)  0 (0 to 0.3) | 0 (0 to 20)  0 (0 to 0.4) | r_pb_: 0.02 (-0.01 to 0.06)  r_pb_: -0.00 (-0.04 to 0.03) | 0.25  0.88 |
| total pH [6.8 to 7[ - s  - % of total time | 0 (0 to 0)  0 (0 to 0) | 0 (0 to 0)  0 (0 to 0) | 0 (0 to 0)  0 (0 to 0) | r_pb_: -0.02 (-0.05 to 0.02)  r_pb_: -0.02 (-0.06 to 0.01) | 0.40  0.22 |
| total pH [7 to 7.2[ - s  - % of total time | 0 (0 to 60)  0 (0 to 0.9) | 0 (0 to 100)  0 (0 to 1.1) | 0 (0 to 60)  0 (0 to 0.9) | r_pb_: 0.00 (-0.03 to 0.04)  r_pb_: -0.01 (-0.04 to 0.03) | 0.93  0.66 |
| total pH [7.2 to 7.4[ - s  - % of total time | 3640 (1550 to 5760)  54.9 (24.1 to 78.1) | 4320 (1555 to 6500)  56.1 (22.9 to 78.0) | 3740 (1550 to 5860)  55.0 (23.8 to 78.1) | r_pb_: 0.07 (0.04 to 0.11)  r_pb_: -0.00 (-0.04 to 0.03) | **<0.0001**  0.91 |
| total pH [7.4 to 7.6[ - s  - % of total time | 2360 (720 to 4390)  34.7 (10.1 to 65.1) | 2820 (805 to 5180)  35.0 (10.1 to 64.8) | 2400 (740 to 4460)  34.7 (10.1 to 65.1) | r_pb_: 0.07 (0.04 to 0.11)  r_pb_: -0.00 (-0.04 to 0.03) | **<0.0001**  0.99 |
| total pH [7.6 and more] - s  - % of total time | 60 (0 to 660)  0.9 (0 to 9.4) | 300 (0 to 720)  3.8 (0 to 9.1) | 80 (0 to 680)  1.1 (0 to 9.4) | r_pb_: 0.05 (0.01 to 0.08)  r_pb_: 0.02 (-0.02 to 0.05) | **0.0053**  0.36 |
| **III.3. Longest contiguous interval within pH range** |  |  |  |  |  |
| cont. pH [up to 6.8[ - s  - % of total time | 0 (0 to 20)  0 (0 to 0.4) | 0 (0 to 20)  0 (0 to 0.3) | 0 (0 to 20)  0 (0 to 0.4) | r_pb_: 0.01 (-0.02 to 0.05)  r_pb_: -0.01 (-0.04 to 0.03) | 0.54  0.66 |
| cont. pH [6.8 to 7[ - s  - % of total time | 0 (0 to 0)  0 (0 to 0) | 0 (0 to 0)  0 (0 to 0) | 0 (0 to 0)  0 (0 to 0) | r_pb_: -0.02 (-0.05 to 0.02)  r_pb_: -0.02 (-0.06 to 0.01) | 0.36  0.19 |
| cont. pH [7 to 7.2[ - s  - % of total time | 0 (0 to 40)  0 (0 to 0.5) | 0 (0 to 60)  0 (0 to 0.6) | 0 (0 to 40)  0 (0 to 0.5) | r_pb_: 0.01 (-0.02 to 0.04)  r_pb_: -0.00 (-0.04 to 0.03) | 0.57  0.87 |
| cont. pH [7.2 to 7.4[ - s  - % of total time | 1980 (820 to 3820)  28.1 (12.2 to 54.1) | 2270 (820 to 4100)  27.1 (11.0 to 49.5) | 2020 (820 to 3860)  27.9 (12.0 to 53.5) | r_pb_: 0.04 (0.00 to 0.07)  r_pb_: -0.02 (-0.06 to 0.01) | **0.040**  0.21 |
| cont. pH [7.4 to 7.6[ - s  - % of total time | 1320 (450 to 2600)  17.7 (6.3 to 38.2) | 1510 (475 to 2980)  18.3 (6.1 to 35.4) | 1340 (460 to 2640)  18.0 (6.3 to 37.9) | r_pb_: 0.05 (0.01 to 0.08)  r_pb_: -0.01 (-0.05 to 0.02) | **0.0079**  0.43 |
| cont. pH [7.6 and more] - s  - % of total time | 40 (0 to 640)  0.7 (0 to 9.1) | 220 (0 to 700)  3.1 (0 to 8.9) | 60 (0 to 660)  1.1 (0 to 9.1) | r_pb_: 0.04 (0.01 to 0.08)  r_pb_: 0.01 (-0.02 to 0.05) | **0.013**  0.46 |
| **IV. PaCO2**  **IV.1. Absolute PaCO2** |  |  |  |  |  |
| median PaCO2 – mmHg | 38 (36 to 40) | 38 (35 to 39) | 38 (36 to 40) | r_pb_: -0.01 (-0.04 to 0.03) | 0.72 |
| minimal PaCO2 – mmHg | 0 (0 to 11) | 0 (0 to 9) | 0 (0 to 11) | r_pb_: -0.02 (-0.06 to 0.01) | 0.19 |
| maximal PaCO2 – mmHg | 54 (44 to 72) | 54 (45 to 72) | 54 (45 to 72) | r_pb_: 0.00 (-0.03 to 0.04) | 0.93 |
| minimal PaCO2 [140 s] – mmHg | 21.9 (0 to 29.4) | 15.4 (0 to 28) | 21.1 (0 to 29.1) | r_pb_: -0.07 (-0.11 to -0.04) | **<0.0001** |
| maximal PaCO2 [140 s] - mmHg | 48.9 (43 to 60.9) | 48.1 (43.4 to 61.3) | 48.7 (43 to 60.9) | r_pb_: -0.01 (-0.05 to 0.02) | 0.47 |
| **IV.2. Cumulative time within PaCO2 range** |  |  |  |  |  |
| total PaCO2 [up to 20 mmHg[ - s  - % of total time | 60 (20 to 580)  0.9 (0.4 to 7.9) | 100 (40 to 680)  1.5 (0.4 to 8.4) | 60 (20 to 600)  0.9 (0.4 to 7.9) | r_pb_: 0.06 (0.03 to 0.10)  r_pb_: 0.03 (-0.00 to 0.07) | **0.0003**  0.063 |
| total PaCO2 [20 to 30 mmHg[ - s  - % of total time | 80 (20 to 320)  1.1 (0.3 to 4.2) | 120 (20 to 440)  1.5 (0.4 to 4.9) | 80 (20 to 340)  1.1 (0.3 to 4.3) | r_pb_: 0.06 (0.02 to 0.09)  r_pb_: 0.02 (-0.02 to 0.05) | **0.0009**  0.37 |
| total PaCO2 [30 to 40 mmHg[ - s  - % of total time | 3840 (2240 to 5450)  58.0 (35.0 to 76.9) | 4680 (2915 to 6305)  60.1 (39.2 to 75.2) | 3960 (2320 to 5560)  58.5 (35.5 to 76.6) | r_pb_: 0.11 (0.08 to 0.15)  r_pb_: 0.02 (-0.02 to 0.05) | **<0.0001**  0.29 |
| total PaCO2 [40 to 50 mmHg[ - s  - % of total time | 1780 (450 to 3540)  25.8 (6.8 to 48.4) | 2010 (440 to 3770)  23.9 (6.4 to 43.1) | 1820 (440 to 3580)  25.4 (6.8 to 47.6) | r_pb_: 0.03 (-0.00 to 0.06)  r_pb_: -0.03 (-0.07 to 0.00) | 0.092  0.078 |
| total PaCO2 [50 to 60 mmHg[ - s  - % of total time | 0 (0 to 180)  0 (0 to 2.1) | 0 (0 to 160)  0 (0 to 1.8) | 0 (0 to 180)  0 (0 to 2.1) | r_pb_: -0.01 (ß0.04 to 0.03)  r_pb_: -0.03 (-0.07 to 0.00) | 0.77  0.077 |
| total PaCO2 [60 mmHg and more] - s  - % of total time | 0 (0 to 220)  0 (0 to 3.0) | 0 (0 to 145)  0 (0 to 1.8) | 0 (0 to 200)  0 (0 to 2.7) | r_pb_: 0.01 (-0.02 to 0.05)  r_pb_: -0.01 (-0.04 to 0.03) | 0.46  0.67 |
| **IV.3. Longest contiguous time interval within PaCO2 range** |  |  |  |  |  |
| cont. PaCO2 [up to 20 mmHg[ - s  - % of total time | 40 (20 to 560)  0.8 (0.4 to 7.5) | 80 (40 to 660)  1.2 (0.4 to 7.9) | 60 (20 to 580)  0.8 (0.4 to 7.6) | r_pb_: 0.07 (0.03 to 0.10)  r_pb_: 0.03 (-0.01 to 0.07) | **0.0002**  0.083 |
| cont. PaCO2 [20 to 30 mmHg[ - s  - % of total time | 60 (20 to 200)  0.7 (0.3 to 2.8) | 80 (20 to 280)  0.9 (0.3 to 2.9) | 60 (20 to 220)  0.8 (0.3 to 2.8) | r_pb_: 0.05 (0.01 to 0.08)  r_pb_: 0.00 (-0.03 to 0.04) | **0.005**  0.91 |
| cont. PaCO2 [30 to 40 mmHg[ - s  - % of total time | 1820 (1070 to 3020)  25.5 (14.7 to 44.1) | 2120 (1335 to 3225)  25.1 (15.4 to 41.8) | 1880 (1110 to 3040)  25.5 (14.8 to 43.7) | r_pb_: 0.05 (0.01 to 0.08)  r_pb_: -0.02 (-0.06 to 0.01) | **0.0072**  0.23 |
| cont. PaCO2 [40 to 50 mmHg[ - s  - % of total time | 760 (260 to 1480)  10.6 (3.4 to 21.3) | 850 (240 to 1460)  10.0 (3.3 to 17.6) | 760 (260 to 1480)  10.4 (3.4 to 20.5) | r_pb_: 0.01 (-0.03 to 0.04)  r_pb_: -0.04 (-0.08 to -0.01) | 0.62  **0.022** |
| cont. PaCO2 [50 to 60 mmHg[ - s  - % of total time | 0 (0 to 80)  0 (0 to 0.9) | 0 (0 to 60)  0 ( 0 to 0.8) | 0 (0 to 80)  0 (0 to 0.9) | r_pb_: -0.02 (-0.05 to 0.02)  r_pb_:-0.05 (-0.08 to -0.01) | 0.30  **0.011** |
| cont. PaCO2 [60 mmHg and more] - s  - % of total time | 0 (0 to 80)  0 (0 to 1.2) | 0 (0 to 80)  0 (0 to 0.8) | 0 (0 to 80)  0 (0 to 1.2) | r_pb_: 0.01 (-0.03 to 0.04)  r_pb_: -0.01 (-0.05 to 0.02) | 0.74  0.50 |
| **V. PaO2**  **V.1. Absolute PaO2** |  |  |  |  |  |
| median PaO2 – mmHg | 240 (221 to 260) | 247 (227 to 267) | 241 (221 to 261) | r_pb_: 0.03 (-0.00 to 0.07) | 0.054 |
| minimal PaO2 – mmHg | 143 (119 o 169) | 147 (121 to 174) | 144 (119 to 170) | r_pb_: 0.02 (-0.02 to 0.06) | 0.40 |
| maximal PaO2 – mmHg | 414 (382 to 444) | 414 (380 to 44) | 414 (382 to 444) | r_pb_: -0.00 (-0.04 to 0.03) | 0.86 |
| minimal PaO2 [140 s] – mmHg | 174 (151 to 195) | 175 (152 to 196) | 174 (151 to 196) | r_pb_: -0.00 (-0.05 to 0.04) | 0.72 |
| maximal PaO2 [140 s] - mmHg | 374 (339 to 411) | 383 (347 to 422) | 375 (340 to 413) | r_pb_: 0.06 (0.02 to 0.09) | **0.0013** |
| **V.2. Cumulative PaO2 time within range** |  |  |  |  |  |
| total PaO2 [up to 70 mmHg[ - s  - % of total time | 0 (0 to 540)  0 (0 to 7.2) | 0 (0 to 620)  0 (0 to 7.6) | 0 (0 to 560)  0 (0 to 7.3) | r_pb_: 0.05 (0.02 to 0.09)  r_pb_: 0.02 (-0.01 to 0.06) | **0.0035**  0.21 |
| total PaO2 [70 to 110 mmHg[ - s  - % of total time | 0 (0 to 0)  0 (0 to 0) | 0 (0 to 0)  0 (0 to 0) | 0 (0 to 0)  0 (0 to 0) | r_pb_: 0.01 (-0.03 to 0.04)  r_pb_: -0.01 (-0.04 to 0.03) | 0.76  0.69 |
| total PaO2 [110 to 140 mmHg[ - s  - % of total time | 0 (0 to 40)  0 (0 to 0.5) | 0 (0 to 20)  0 (0 to 0.3) | 0 (0 to 40)  0 (0 to 0.5) | r_pb_: -0.03 (-0.07 to 0.00)  r_pb_: -0.05 (-0.09 to -0.02) | 0.069  **0.0037** |
| total PaO2 [140 to 190 mmHg[ - s  - % of total time | 240 (40 to 820)  3.3 (0.7 to 10.9) | 180 (20 to 680)  2.3 (0.3 to 7.5) | 240 (40 to 780)  3.1 (0.6 to 10.5) | r_pb_: -0.03 (-0.06 to 0.01)  r_pb_: -0.07 (-0.10 to -0.03) | 0.12  **0.0003** |
| total PaO2 [190 to 240 mmHg[ - s  - % of total time | 2360 (1180 to 3700)  34.9 (17.4 to 52.3) | 2320 (1200 to 3765)  29.8 (15.1 to 48.5) | 2360 (1180 to 3720)  34.1 (17.0 to 51.7) | r_pb_: 0.02 (-0.01 to 0.06)  r_pb_: -0.05 (-0.09 to -0.02) | 0.18  **0.0037** |
| total PaO2 [240 mmHg and more] - s  - % of total time | 3300 (1860 to 5190)  50.0 (29.6 to 72.6) | 4260 (2575 to 6730)  59.2 (36.5 to 77.2) | 3420 (1940 to 5400)  51.2 (30.3 to 73.3) | r_pb_: 0.13 (0.10 to0.17)  r_pb_: 0.06 (0.03 to 0.10) | **<0.0001**  **0.0004** |
| **V.3. Longest contiguous time interval within PaO2 range** |  |  |  |  |  |
| cont. PaO2 [up to 70 mmHg[ - s  - % of total time | 0 (0 to 540)  0 (0 to 7.1) | 0 (0 to 620)  0 (0 to 7.4) | 0 (0 to 540)  0 (0 to 7.2) | r_pb_: 0.05 (0.02 to 0.09)  r_pb_: 0.02 (-0.01 to 0.06) | **0.0033**  0.26 |
| cont. PaO2 [70 to 110 mmHg[ - s  - % of total time | 0 (0 to 0)  0 (0 to 0) | 0 (0 to 0)  0 (0 to 0) | 0 (0 to 0)  0 (0 to 0) | r_pb_: 0.00 (-0.03 to 0.04)  r_pb_: -0.01 (-0.05 to 0.02) | 0.98  0.43 |
| cont. PaO2 [110 to 140 mmHg[ - s  - % of total time | 0 (0 to 20)  0 (0 to 0.4) | 0 (0 to 20)  0 (0 to 0.3) | 0 (0 to 20)  0 (0 to 0.4) | r_pb_: -0.04 (-0.07 to -0.00)  r_pb_: -0.05 (-0.09 to -0.02) | **0.036**  **0.0038** |
| cont. PaO2 [140 to 190 mmHg[ - s  - % of total time | 80 (40 to 300)  1.2 (0.5 to 3.9) | 80 (20 to 240)  0.9 (0.2 to 2.8) | 80 (40 to 280)  1.2 (0.4 to 3.7) | r_pb_: -0.02 (-0.06 to 0.01)  r_pb_: -0.06 (-0.09 to -0.02) | 0.20  **0.0016** |
| cont. PaO2 [190 to 240 mmHg[ - s  - % of total time | 740 (360 to 1200)  10.6 (5.1 to 17.1) | 780 (360 to 1185)  9.3 (4.0 to 16.2) | 740 (360 to 1200)  10.4 (4.9 to 17.0) | r_pb_: 0.01 (-0.03 to 0.04)  r_pb_: -0.04 (-0.08 to -0.01) | 0.61  **0.014** |
| cont. PaO2 [240 mmHg and more] - s  - % of total time | 1420 (820 to 2670)  21.5 (12.2 to 37.6) | 2040 (1100 to 3540)  25.7 (15.7 to 44.1) | 1520 (860 to 2810)  22.3 (12.5 to 38.6) | r_pb_: 0.13 (0.09 to 0.16)  r_pb_: 0.06 (0.03 to 0.10) | **<0.0001**  **0.0007** |
| **VI. Blood temperature**  **VI.1. Absolute temperature** |  |  |  |  |  |
| median temperature – °C | 35.7 (34.7 to 36.1) | 35.6 (33.6 to 36.0) | 35.7 (34.6 to 36.1) | r_pb_: -0.01 (-0.04 to 0.03) | 0.61 |
| minimal temperature – °C | 33.4 (30.5 to 34.8) | 32.7 (28.8 to 34.8) | 33.3 (30.0 to 34.8) | r_pb_: -0.08 (-0.11 to -0.04) | **<0.0001** |
| maximal temperature – °C | 36.5 (35.9 to 37) | 36.5 (35.8 to 37.1) | 36.5 (35.9 to 37.0) | r_pb_: -0.03 (-0.07 to 0.00) | 0.056 |
| minimal temperature [140 s] – °C | 34.5 (32.5 to 35.3) | 33.8 (30.0 to 35.2) | 34.4 (32.2 to 35.3) | r_pb_: -0.10 (-0.14 to -0.07) | **<0.0001** |
| maximal temperature [140 s] - °C | 36.5 (35.9 to 37.0) | 36.4 (35.8 to 37.0) | 36.5 (35.9 to 37.0) | r_pb_: -0.04 (-0.07 to -0.00) | **0.040** |
| **VI.2. Cumulative time within temperature range** |  |  |  |  |  |
| total temperature [up to 31 °C[ - s  - % of total time | 0 (0 to 20)  0 (0 to 0.2) | 0 (0 to 65)  0 (0 to 1.6) | 0 (0 to 20)  0 (0 to 0.3) | r_pb_: 0.09 (0.05 to 0.12)  r_pb_: 0.07 (0.04 to 0.11) | **<0.0001**  **<0.0001** |
| total temperature [31 to 33 °C[ - s  - % of total time | 0 (0 to 40)  0 (0 to 0.6) | 0 (0 to 165)  0 (0 to 2.2) | 0 (0 to 40)  0 (0 to 0.7) | r_pb_: 0.06 (0.03 to 0.09)  r_pb_: 0.04 (0.01 to 0.08) | **0.0007**  **0.025** |
| total temperature [33 to 35 °C[ - s  - % of total time | 80 (0 to 1060)  1.5 (0 to 16.9) | 120 (0 to 1315)  1.8 (0 to 18.0) | 80 (0 to 1080)  1.6 (0 to 16.9) | r_pb_: 0.03 (-0.00 to 0.07)  r_pb_: 0.01 (-0.02 to 0.05) | 0.051  0.49 |
| total temperature [35 to 37 °C[ - s  - % of total time | 4900 (2140 to 6740)  88.4 (39.7 to 99.5) | 4640 (1260 to 7265)  75.8 (23.2 to 99.3) | 4880 (2020 to 6800)  86.3 (36.9 to 99.5) | r_pb_: 0.01 (-0.02 to 0.05)  r_pb_: -0.07 (-0.11 to -0.04) | 0.47  **0.0001** |
| total temperature [37 to 39°C mmHg[ - s  - % of total time | 0 (0 to 20)  0 (0 to 1.4) | 0 (0 to 100)  0 (0 to 2.3) | 0 (0 to 40)  0 (0 to 1.5) | r_pb_: 0.03 (-0.01 to 0.06)  r_pb_: 0.01 (-0.03 to 0.04) | 0.12  0.71 |
| total temperature [39 °C and more] - s  - % of total time | 0 (0 to 0)  0 (0 to 0) | 0 (0 to 0)  0 (0 to 0) | 0 (0 to 0)  0 (0 to 0) | r_pb_: 0.02 (-0.02 to 0.05)  r_pb_: 0.03 (-0.01 to 0.06) | 0.30  0.12 |
| **VI.3. Longest contiguous time interval within temperature range** |  |  |  |  |  |
| cont. temperature [up to 31 °C[ - s  - % of total time | 0 (0 to 20)  0 (0 to 0.2) | 0 (0 to 60)  0 (0 to 1.1) | 0 (0 to 20)  0 (0 to 0.3) | r_pb_: 0.09 (0.05 to 0.12)  r_pb_: 0.07 (0.04 to 0.11) | **<0.0001**  **<0.0001** |
| cont. temperature [31 to 33 °C[ - s  - % of total time | 0 (0 to 20)  0 (0 to 0.5) | 0 (0 to 120)  0 (0 to 1.6) | 0 (0 to 40)  0 (0 to 0.6) | r_pb_: 0.06 (0.03 to 0.10)  r_pb_: 0.05 (0.01 to 0.08) | **0.0004**  **0.014** |
| cont. temperature [33 to 35 °C[ - s  - % of total time | 60 (0 to 720)  1.3 (0 to 11.1) | 100 (0 to 885)  1.3 (0 to 12.1) | 80 (0 to 740)  1.3 (0 to 11.1) | r_pb_: 0.03 (-0.00 to 0.07)  r_pb_: 0.01 (-0.02 to 0.05) | 0.050  0.45 |
| cont. temperature [35 to 37 °C[ - s  - % of total time | 3980 (1400 to 6080)  67.1 (24.6 to 99.4) | 3590 (860 to 6485)  52.4 (13.5 to 99.1) | 3940 (1320 to 6120)  65.4 (23.0 to 99.4) | r_pb_: 0.01 (-0.02 to 0.05)  r_pb_: -0.06 (-0.10 to -0.03) | 0.53  **0.0008** |
| cont. temperature [37 to 39 °C[ - s  - % of total time | 0 (0 to 20)  0 (0 to 0.9) | 0 (0 to 85)  0 (0 to 2.3) | 0 (0 to 30)  0 (0 to0.94 | r_pb_: 0.03 (-0.00 to 0.07)  r_pb_: 0.01 (-0.03 to 0.04) | 0.056  0.63 |
| cont. temperature [39°C and more] - s  - % of total time | 0 (0 to 0)  0 (0 to 0) | 0 (0 to 0)  0 (0 to 0) | 0 (0 to 0)  0 (0 to 0) | r_pb_: 0.02 (-0.02 to 0.05)  r_pb_: 0.03 (-0.01 to 0.06) | 0.37  0.16 |
| **VII. ‍Hematocrit**  **VII.1. Absolute hematocrit** |  |  |  |  |  |
| median Hct – % | 34.8 (32.83 to 35.70) | 34.6 (31.38 to 35.60) | 34.8 (32.70 to 35.70) | r_pb_: -0.00 (-0.04 to 0.03) | 0.78 |
| minimal Hct – % | 32.10 (27.50 to 34.00) | 31.20 (25.58 to 33.70) | 32.00 (27.20 to 33.90) | r_pb_: -0.02 (-0.06 to 0.01) | 0.20 |
| maximal Hct – % | 35.70 (34.40 to 36.40) | 35.70 (34.08 to 36.40) | 35.70 (34.40 to 36.40) | r_pb_: 0.01 (-0.03 to 0.04) | 0.67 |
| minimal Hct [140 s] – % | 33.17 (29.87 to 34.40) | 32.67 (26.31 to 34.16) | 33.09 (29.42 to 34.37) | r_pb_: -0.03 (-0.06 to 0.01) | 0.13 |
| maximal Hct [140 s] – % | 35.70 (34.39 to 36.39) | 35.70 (34.06 to 36.30) | 35.70 (34.31 to 36.37) | r_pb_: 0.01 (-0.03 to 0.04) | 0.66 |
| **VII.2. Cumulative time within hematocrit range** |  |  |  |  |  |
| Hct [up to 20%[ – s | 0 (0 to 0) | 0 (0 to 0) | 0 (0 to 0) | r_pb_: 0.03 (-0.00 to 0.07) | 0.083 |
| **Hct [20% to 30%[ – s** | **0 (0 to 20)** | **0 (0 to 145)** | **0 (0 to 20)** | **r_pb_: 0.07 (0.03 to 0.10)** | **0.0002** |
| **Hct [30% to 40%[ – s** | **6060 (4300 to 7730)** | **6680 (4700 to 8720)** | **6120 (4340 to 7900)** | **r_pb_: 0.07 (0.03 to 0.10)** | **0.0002** |
| ‍Hct [40% and more] – s | 0 (0 to 0) | 0 (0 to 0) | 0 (0 to 0) | r_pb_: 0.03 (-0.01 to 0.06) | 0.15 |
| ‍VII.3. **Longest contiguous time interval within hematocrit range** |  |  |  |  |  |
| ‍cont. Hct [up to 20%[ – s | 0 (0 to 0) | 0 (0 to 0) | 0 (0 to 0) | r_pb_: 0.03 (-0.01 to 0.06) | 0.10 |
| **cont. Hct [20% to 30%[ – s** | **0 (0 to 20)** | **0 (0 to 100)** | **0 (0 to 20)** | **r_pb_: 0.07 (0.03 to 0.10)** | **0.0001** |
| **cont. Hct [30% to 40%[ – s** | **5940 (4170 to 7600)** | **6470 (4475 to 8525)** | **6000 (4200 to 7700)** | **r_pb_: 0.06 (0.03 to 0.09)** | **0.0007** |
| ‍cont. Hct [40% and more] – s | 0 (0 to 0) | 0 (0 to 0) | 0 (0 to 0) | r_pb_: 0.03 (-0.01 to 0.06) | 0.15 |

Continuous numeric data are presented as mean (1^st^ quartile to 3^rd^ quartile); r_pb_, point-biserial correlation coefficient; CI, 95% confidence interval.

Cont. = contiguous

Supplemental table 7: Predictors of postoperative delirium as identified by multiple imputation and multivariable logistic regression.

| factor | β (95% confidence interval) | adjusted odds ratio (95% confidence interval) |
| --- | --- | --- |
| (Intercept) | -3.616 (CI: -4.718 to -2.514) | 0 (CI: 0 to 0) |
| CPB system | 1.030 (CI: -0.028 to 2.087) | 2.800 (CI: 0.972 to 8.063) |
| **status post reanimationacute renal failure** | **0.650 (CI: 0.329 to 0.972)** | **1.916 (CI: 1.390 to 2.643)** |
| **male sex** | **0.633 (CI: 0.332 to 0.935)** | **1.884 (CI: 1.393 to 2.547)** |
| **age (y)** | **0.599 (CI: 0.466 to 0.732)** | **1.057 (CI: 1.044 to 1.070)** |
| **diabetes mellitus** | **0.488 (CI: 0.244 to 0.733)** | **1.630 (CI: 1.276 to 2.081)** |
| **carotid disease** | **0.405 (CI: 0.050 to 0.760)** | **1.499 (CI: 1.051 to 2.138)** |
| chronic renal failure | 0.325 (CI: -0.015 to 0.664) | 1.383 (CI: 0.985 to 1.942) |
| **total CPB blood volume (L)** | **0.310 (CI: 0.175 to 0.446)** | **1.001 (CI: 1.001 to 1.002)** |
| non-elective surgery**year** | **0.150 (CI: 0.038 to 0.263)** | **1.084 (CI: 1.020 to 1.151)** |
| ‍longest contiguous time interval at > 30% above target CPB blood flow (h) | 0.086 (CI: -0.007 to 0.178) | **2.359**1.819 (CI: 0.955 to 3.463) |
| ‍non-elective surgery | 0.092 (CI: -0.012 to 0.196) | 0.9981.135 (CI: 0.984 to 1.310) |
| hematocrit abs. 30% incl. to 40% excl. total (h) | -0.107 (CI: -0.231 to 0.017) | 0.898 (CI: 0.793 to 1.017) |
| ‍cardioplegia set | -0.157 (CI: -0.397 to 0.083) | 0.854 (CI: 0.672 to 1.086) |
| longest contiguous time interval at 110 mmHg ≤ PaO_2_ < 140 mmHg (h) | -0.115 (CI: -0.257 to 0.026) | 2.537E-4 (CI:9.580E-9 to 6.720) |
| **‍target flow (L·min^-1^)** | **-0.157 (CI: -0.294 to -0.021)** | **0.724 (CI: 0.547 to 0.958)** |
| **‍minimum flow relative to target [140 s] (L·min^-1^)** | **-0.189 (CI: -0.304 to -0.074)** | **0.993 (CI: 0.988 to 0.997)** |

Pooled beta values and exponentiated pooled point estimators of the minimal adequate model calculated from 10 imputations (n=3163 patients).

CBP = cardiopulmonary bypass

Supplemental table 8: Missingness of imputed variables

| factor | missing values – n (%) |
| --- | --- |
| age on entry | 0 (0) |
| male sex | 0 (0) |
| acute kidney failure | 0 (0) |
| chronic kidney failure | 0 (0) |
| carotid disease | 0 (0) |
| blood transfusion | 0 (0) |
| arterial hypertension | 0 (0) |
| hyperlipoproteinemia | 0 (0) |
| atrial fibrillation | 0 (0) |
| diabetes mellitus | 0 (0) |
| right heart failure | 0 (0) |
| history of cardiopulmonary resuscitation | 0 (0) |
| emergency surgery | 1 (0.03%) |
| year of surgery | 0 (0) |
| oxygenator | 2 (0.06%) |
| reservoir | 209 (6.6%) |
| CPB system | 0 (0) |
| vent location | 53 (1.7%) |
| cardioplegia set | 56 (1.8%) |
| tub pack | 86 (2.7%) |
| type of surgery | 0 (0) |
| deep cooling | 0 (0) |
| selective cerebral perfusion | 0 (0) |
| target flow | 0 (0) |
| lowest CPB blood flow relative windowed for 140 sec. | 0 (0) |
| CBP blood flow abs. 0 incl. to 0.75 L·min^-1^·m^-2^excl. total | 5 (0.2%) |
| CBP blood flow abs. 3.0 incl. to 3.75 L·min^-1^·m^-2^ excl. total | 5 (0.2%) |
| CBP blood flow deviation to target value 30% incl. to inf excl. cont. | 0 (0) |
| patient blood pressure minus inf incl. to 40 mmHg excl. total | 0 (0) |
| patient blood pressure 40 incl. to 60 mmHg excl. total | 0 (0) |
| pH art. 7.2 incl. to 7.4 excl. total | 0 (0) |
| pH art. 7.6 incl. to inf excl. total | 0 (0) |
| pCO_2_ art. 0 incl. to 20 mmHg excl. total | 0 (0) |
| pCO_2_ art. 20 incl. to 30 mmHg excl. total | 0 (0) |
| pCO_2_ art. 20 incl. to 30 mmHg excl. cont. | 0 (0) |
| pCO_2_ art. 30 incl. to 40 mmHg excl. total | 0 (0) |
| pO_2_ art. 30 incl. to 40 mmHg excl. cont. | 0 (0) |
| pO_2_ art. 110 incl. to 140 mmHg excl. cont. | 0 (0) |
| pO_2_ art. 240 mmHg incl. to inf excl. total | 0 (0) |
| Hct 20% incl. to 30% excl | 0 (0) |
| Hct 30% incl. to 40% excl | 0 (0) |
| temperature minus inf incl. to 31 excl. total | 0 (0) |
| temperature 31°C incl. to 33°C excl. total | 0 (0) |
| total CBP blood volume | 62 (2.0%) |
| aortic cross-clamp time | 0 |
| reperfusion time | 0 |
| lowest total temperature | 217 (6.9%) |

Supplemental table 9: Combinations and Frequencies of CPB Equipment Utilization.

The first row indicates tubing sets. The first column specifies the oxygenators, and the second column the reservoirs.

Dideco Eurosets HMT LivaNova Maquet Medos Medtronic Sorin

Euroset_Skipper Euroset_Admicard_3200 0 2 0 0 0 0 0 0

Euroset_Admiral 0 10 0 0 0 0 0 0

Euroset_Horizon 0 0 0 0 0 0 0 0

LivaNova_Inspire 0 0 0 0 0 0 0 0

Maquet_VHK_7100 0 0 0 0 5 0 0 0

Medos_Reservoir 0 0 0 0 0 0 0 0

Sorin_D744 2 1 1 0 1 0 0 0

Sorin_Synthesis_R 2 0 0 0 1 0 0 0

Hilite_7000 Euroset_Admicard_3200 0 0 0 0 0 0 0 0

Euroset_Admiral 0 0 0 0 0 0 0 0

Euroset_Horizon 0 0 0 0 0 0 0 0

LivaNova_Inspire 0 0 0 0 0 0 0 0

Maquet_VHK_7100 0 0 0 0 3 5 0 0

Medos_Reservoir 0 0 2 0 2 137 0 0

Sorin_D744 0 0 0 0 0 1 0 0

Sorin_Synthesis_R 2 0 1 0 2 4 0 0

Maquet_Quadrox Euroset_Admicard_3200 0 7 0 0 2 0 0 0

Euroset_Admiral 0 120 1 1 67 0 0 0

Euroset_Horizon 0 0 0 0 0 0 0 0

LivaNova_Inspire 0 0 0 3 0 0 0 0

Maquet_VHK_7100 0 31 12 9 140 0 0 1

Medos_Reservoir 0 0 0 0 0 0 0 0

Sorin_D744 291 3 242 3 2 1 0 5

Sorin_Synthesis_R 1 0 0 1 0 0 0 0

Medtronic_Fusion Euroset_Admicard_3200 0 1 0 0 0 0 0 0

Euroset_Admiral 0 11 6 3 0 0 2 0

Euroset_Horizon 0 0 1 4 0 0 0 0

LivaNova_Inspire 0 0 0 0 0 0 0 0

Maquet_VHK_7100 0 0 3 0 1 0 0 0

Medos_Reservoir 0 0 0 0 0 0 0 0

Sorin_D744 0 3 137 5 0 0 1 1

Sorin_Synthesis_R 0 0 0 0 0 0 0 0

Paragon Euroset_Admicard_3200 0 0 0 0 0 0 0 0

Euroset_Admiral 0 0 0 0 0 0 0 0

Euroset_Horizon 0 0 0 0 0 0 0 0

LivaNova_Inspire 0 0 0 0 0 0 0 0

Maquet_VHK_7100 0 0 0 0 2 0 0 0

Medos_Reservoir 0 0 0 0 0 0 0 0

Sorin_D744 2 0 0 0 0 0 0 0

Sorin_Synthesis_R 0 0 0 0 0 0 0 0

Sorin_Inspire Euroset_Admicard_3200 0 68 0 2 0 0 0 0

Euroset_Admiral 1 203 19 9 0 0 0 0

Euroset_Horizon 0 10 0 2 0 0 0 0

LivaNova_Inspire 0 0 0 13 0 0 0 0

Maquet_VHK_7100 6 0 0 0 21 0 0 1

Medos_Reservoir 0 0 0 0 0 3 0 0

Sorin_D744 25 36 247 8 0 0 0 9

Sorin_Synthesis_R 310 15 74 12 201 4 0 8

Sorin_Synthesis Euroset_Admicard_3200 0 0 0 0 0 0 0 0

Euroset_Admiral 0 0 0 0 0 0 0 0

Euroset_Horizon 0 0 0 0 0 0 0 0

LivaNova_Inspire 0 0 0 0 0 0 0 0

Maquet_VHK_7100 0 0 0 0 11 0 0 0

Medos_Reservoir 0 0 0 0 0 0 0 0

Sorin_D744 15 0 0 0 1 0 0 0

Sorin_Synthesis_R 1 0 0 1 0 0 0 0

Terumo_Capiox Euroset_Admicard_3200 0 0 0 0 0 0 0 0

Euroset_Admiral 0 0 0 0 0 0 0 0

Euroset_Horizon 0 0 0 0 0 0 0 0

LivaNova_Inspire 0 0 0 0 0 0 0 0

Maquet_VHK_7100 2 0 3 0 203 0 0 0

Medos_Reservoir 0 0 0 0 0 0 0 0

Sorin_D744 6 0 4 0 0 0 0 1

Sorin_Synthesis_R 3 0 6 0 0 0 0 0
